# Exponential equilibration of genetic circuits using entropy methods

**DOI:** 10.1007/s00285-018-1277-z

**Published:** 2018-08-17

**Authors:** José A. Cañizo, José A. Carrillo, Manuel Pájaro

**Affiliations:** 1 Departamento de Matemática Aplicada, Universidad de Granada, 18071 Granada, Spain; 2 Department of Mathematics, Imperial College London, London, SW7 2AZ UK; 3 BioProcess Engineering Group, IIM-CSIC, Spanish Council for Scientific Research, Eduardo Cabello 6, 36208 Vigo, Spain

**Keywords:** 35B40, 92Dxx, 39B99, 65M99

## Abstract

We analyse a continuum model for genetic circuits based on a partial integro-differential equation initially proposed in Friedman et al. (Phys Rev Lett 97(16):168302, 2006) as an approximation of a chemical master equation. We use entropy methods to show exponentially fast convergence to equilibrium for this model with explicit bounds. The asymptotic equilibration for the multidimensional case of more than one gene is also obtained under suitable assumptions on the equilibrium stationary states. The asymptotic equilibration property for networks involving one and more than one gene is investigated via numerical simulations.

## Introduction

Translation of the information encoded in genes is responsible for all cellular functions. The decoding of DNA can be summarised, following the central dogma of molecular biology, in two steps: the transcription into messenger RNA and the translation into proteins. Cells produce responses to environmental signals, thanks to the regulation of DNA expression via certain feedback mechanism activating or inhibiting the genes. Typically, regulation is produced by the union of proteins to the DNA binding sites. Moreover, the number of species involved in gene regulatory networks (gene expression together with their regulation) is small, which makes its behaviour inherently stochastic (Elowitz et al. 2002; Gillespie 2007; Kaeet al. 2005; McAdams and Arkin 1997; Paulsson 2004). This underlying stochastic behaviour in gene regulatory networks is captured by using the chemical master equation (CME) (Kepler and Elston 2001; Mackey et al. 2011; Paulsson 2005; Sherman and Cohen 2014). However, the CME solution is unavailable in most cases, due to the large (even infinite) number of coupled equations.

There are two main ways to obtain the CME solution: via stochastic simulation or via approximations of the CME. One of the most extended methods to reproduce the CME dynamics using stochastic realisations is the stochastic simulation algorithm (SSA) (Gillespie 1976, 2007). This method has no restrictions in its applicability, even though it is computationally expensive. On the other hand, CME approximations which remain valid under certain conditions include the finite state projection (Munsky and Khammash 2006), moment methods (Engblom 2006; Hasenauer et al. 2015), linear noise approximations (Thomas et al. 2014; Kampen 2007; Wallace et al. 2012) or hybrid models (Jahnke 2011).

In addition to the above mentioned methods, assuming that protein production takes place in bursts one can obtain a partial integro-differential equation (PIDE) as a continuous approximation of the CME. This PIDE has a mathematical structure very similar to kinetic and transport equations in mathematical biology (Perthame 2007) and it admits an analytical solution for its steady state in the case of networks involving only one gene. In the next subsections, we describe both the one dimensional PIDE model (Friedman et al. 2006) for self-regulated gene networks and the generalised PIDE model (Pájaro et al. 2017) for arbitrary genetic circuits. We will discuss the main properties of the stationary states in one dimension to finally explain the main results of this work.

### 1-dimensional PIDE model

**Fig. 1:**
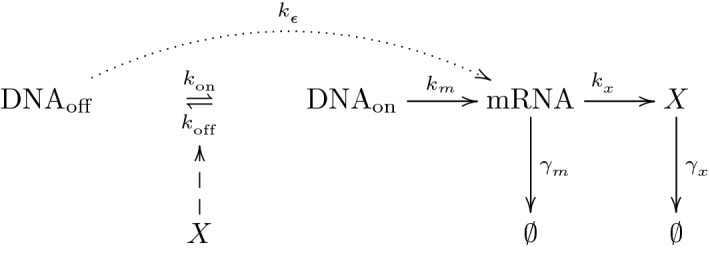
Schematic representation of the transcription-translation mechanism under study. The promoters associated with the gene of interest are assumed to switch between active (DNA$_\mathrm{on}$) and inactive (DNA$_\mathrm{off}$) states, with rate constants $k_{\mathrm {on}}$ and $k_{\mathrm {off}}$ per unit time, respectively. In this study, the transition is assumed to be controlled by a feedback mechanism induced by the binding/unbinding of a given number of *X*-protein molecules, what makes the network self-regulated. Transcription of messenger RNA (mRNA) from the active DNA form, and translation into protein *X* are assumed to occur at rates (per unit time) $k_m$ and $k_x$, respectively. $k_{\varepsilon }$ is the rate constant associated with transcriptional leakage. The mRNA and protein degradations are assumed to occur by first order processes with rate constants ${\gamma }_m$ and ${\gamma }_x$, respectively

The kinetic equation, first proposed by Friedman et al. (2006), is a continuous approximation of the CME for gene self-regulatory networks. A schematic representation of this genetic circuit is illustrated in Fig. 1, where the transcription-translation mechanism from DNA to a protein *X* is shown. Note that DNA transcribes into messenger RNA not only from the active state at rate (per unit time $\tau$) $k_m$, but also from the inactive state with rate constant $k_{\varepsilon }$ lower than $k_m$, which is known as *basal transcription level* or *transcriptional leakage* (Friedman et al. 2006; Ochab-Marcinek and Tabaka 2015; Pájaro et al. 2015). The messenger RNA transcribes into protein *X* following a first-order process with rate constant (per unit time) $k_x$. The messenger RNA and protein are degraded at rate constants $\gamma _m$ and $\gamma _x$ respectively.

For self-regulated gene networks, activation or inhibition of the DNA promoter is produced by the union of the protein expressed to the DNA binding sites (feedback mechanism). So that, under protein action the promoter can switch between its inactive (DNA$_\mathrm{off}$) and active (DNA$_\mathrm{on}$) forms, with rate constants $k_{\mathrm {on}}$ and $k_{\text {off}}$ respectively (see Fig. 1). There are two types of feedback mechanism: positive or negative, corresponding to whether the protein inhibits or promotes their production, respectively. The fraction of the promoter in the active or inactive state is typically described by Hill functions (Alon 2007). We can express the probability that the promoter is in its inactive state as a function of the protein amount *x*, denoted by $\rho :{{\mathbb {R}}}_{+} \rightarrow [0, \ 1]$ (see Ochab-Marcinek and Tabaka 2015; Pájaro et al. 2015):

1.1 $$\begin{aligned} \rho (x)=\dfrac{x^H}{x^H+K^H}, \end{aligned}$$

where $K := \frac{k_{\text {off}}}{k_{\text {on}}}$ is the equilibrium binding constant and $H\in {\mathbb {Z}}\backslash \{0\}$ is the Hill coefficient which is positive if *H* proteins bound to the DNA inhibiting their production (negative feedback) and negative if |*H*| proteins bound to the DNA activating their production (positive feedback). Then, the rate $R_T$ of messenger RNA production (transcription) can be written as function of the Hill expression (1.1), $R_T=k_m c(x)$, with the input function $c(x):= \left( 1 - \rho (x)\right) + \rho (x)\varepsilon$, where $\varepsilon$ is the leakage constant defined as $\varepsilon :=\frac{k_{\varepsilon }}{k_m}$. Note that the function $R_T$ accounts for the messenger RNA production both from the DNA active state (with probability $1 - \rho (x)$) with rate constant $k_m$ and from the inactive DNA (with probability $\rho (x)$) with lower rate constant $k_{\varepsilon }$.

The PIDE model is valid under the assumption of protein production in bursts. So, we consider gene self-regulatory networks where the degradation rate of *mRNA* is much faster than the corresponding to protein, ${\gamma }_m / {\gamma }_x \gg 1$. Such condition is verified in many gene regulatory networks, both in prokaryotic and eukaryotic organisms (Shahrezaei and Swain 2008; Dar et al. 2012), and results in protein being produced in bursts. As suggested in Friedman et al. (2006) and Elgart et al. (2011), the burst size (denoted by $b=\frac{k_x}{\gamma _m}$) is typically modelled by an exponential distribution. The conditional probability for protein level to jump from a state *y* to a state $x > y$ after a burst is proportional to:

1.2 $$\begin{aligned} \omega (x-y)=\dfrac{1}{b}\exp \left( -\dfrac{x-y}{b}\right) , \qquad \text {for}~ x> y > 0. \end{aligned}$$

The temporal evolution of the probability density function of the amount of proteins, $p:{{\mathbb {R}}}_{+}\times {{\mathbb {R}}}_{+} \rightarrow {{\mathbb {R}}}_{+}$ is described by the following PIDE model:

1.3 $$\begin{aligned} \dfrac{\partial p}{\partial t}(t, x) - \dfrac{\partial (xp)}{\partial x}(t, x) = a \int _0^x \! \omega (x-y)c(y)p(t,y) \, \mathrm {d}y - ac(x)p(t,x), \end{aligned}$$

where $\tau$ is time, $t = {\gamma }_x \tau$ represents a dimensionless time associated to the time scale of protein degradation, $a=\frac{k_m}{\gamma _x}$ is the dimensionless rate constant related to transcription, which represents the mean number of bursts (burst frequency) and $\omega (x-y)$ is given by (1.2). The input function $c:{{\mathbb {R}}}_{+} \rightarrow [\varepsilon , \ 1]$, which represents the feedback mechanism, takes the form (Ochab-Marcinek and Tabaka 2015; Pájaro et al. 2015):

1.4 $$\begin{aligned} c(x)=\frac{K^H+ \varepsilon x^H}{K^H + x^H}, \qquad x > 0. \end{aligned}$$

Note that the above input function can be constant, equal to one, when the protein does not promote or repress its production (open loop). This constant $c(x)=1$ is used when the DNA is always in its active state, thus implying a unique messenger RNA production rate ($k_m$), reducing the system complexity.

We denote the *stationary solution* of Eq. (1.3) (which we sometimes call *equilibrium*) as $P_{\infty }(x)$, which therefore verifies the following equation:

1.5 $$\begin{aligned} \dfrac{\partial [xP_{\infty }(x)]}{\partial x} = -a \int _0^x \omega (x-y)c(y)P_{\infty }(y) \mathrm {d}y + ac(x)P_{\infty }(x). \end{aligned}$$

We say a stationary solution is normalised when its integral over $[0,+\infty )$ (which we sometimes call its *mass*) is equal to 1. This equation has a unique solution with mass 1, which can be written out explicitly as (Ochab-Marcinek and Tabaka 2015; Pájaro et al. 2015):

1.6 $$\begin{aligned} P_{\infty }(x):= Z \left[ \rho (x) \right] ^{\frac{a(1-\varepsilon )}{H}} x^{-(1-a\varepsilon )}e^{\frac{-x}{b}} = Z\left[ x^H +K^H \right] ^{\frac{a(\varepsilon -1)}{H}} x^{a-1}e^{\frac{-x}{b}}, \end{aligned}$$

with $\rho (x)$ defined in (1.1) and *Z* being a normalising constant such that $\int _{0}^{\infty }P_{\infty }(x)\, \mathrm {d}x=1$. Alternatively, stationary solutions may be studied by considering the zero-flux case; see for example Bokes and Singh (May 2017); Bokes et al. (Jul 2018).

In case of no self-regulation (open loop network with $c(x)=1$; that is, $\epsilon = 1$) the stationary solution is a gamma distribution (Friedman et al. 2006), which is in fact the limit of (1.6) as $\epsilon$ tends to 1:

1.7 $$\begin{aligned} P_{\infty }(x):=\dfrac{x^{a-1}e^{-x/b}}{b^a \Gamma (a)}. \end{aligned}$$

### Generalised *n*-dimensional PIDE model

Recently the 1D PIDE model has been extended to overcome more general gene regulatory networks than the self-regulation considered by Friedman et al. (2006). As a first step in this extension, Bokes and Singh (2015) propose the use of variable protein degradation rate, in order to accommodate gene networks with decoy binding sites (Lee and Maheshri 2012) to the PIDE model structure. Finally, including the previous models and considering genetic networks involving more than one gene Pájaro et al. (2017) proposed the generalised PIDE model for any number of genes.

**Fig. 2:**
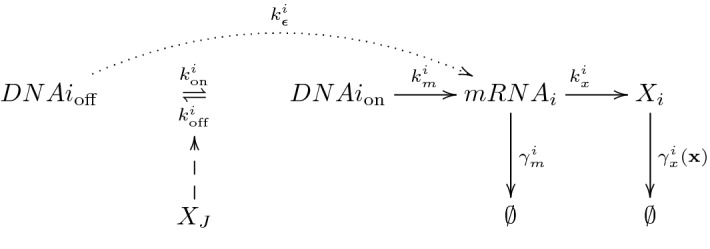
Schematic representation of the transcription-translation mechanism under study. The promoters associated with the genes of interest are assumed to switch between active ($DNAi_{\mathrm {on}}$) and inactive ($DNAi_{\mathrm {off}}$) states, with rate constants $k_{\mathrm {on}}^{i}$ and $k_{\mathrm {off}}^{i}$ per unit time, respectively. The transition is assumed to be controlled by a feedback mechanism induced by the binding/unbinding of a given number of $X_j$-protein molecules with $j \in J$ (more than one protein type can bind to the DNA), which makes the network self-regulated if $i=j$ or cross-regulated if $j\ne i$. Transcription of messenger RNA ($mRNA_i$) from the active *DNAi* form, and translation into protein $X_i$ are assumed to occur at rates (per unit time) $k_m^{i}$ and $k_x^{i}$, respectively. $k_{\varepsilon }^{i}$ is the rate constant associated with transcriptional leakage. The $mRNA_i$ degradation is assumed to occur by first order processes with rate constant ${\gamma }_m^{i}$. Degradation of the $X_i$-protein may follow different pathways, which is modelled by the function ${\gamma }_x^{i} ({\mathbf {x}})$, with $\gamma _x^{i}:{{\mathbb {R}}}_{+}^{n} \rightarrow {{\mathbb {R}}}_{+}$

In Pájaro et al. (2017) a general gene regulatory network comprising *n* genes, $${\varvec{G}}=\{DNA_1, \ldots , DNA_i, \ldots , DNA_n\}$$, is proposed. These genes encoded by DNA-subchains are transcribed into *n* different messenger RNAs $${\varvec{M}}=\{mRNA_1,$$$$ \ldots , mRNA_i, \ldots , mRNA_n\}$$, which are translated into *n* proteins types $${\varvec{X}}=\{X_1, \ldots ,$$$$ X_i, \ldots , X_n\}$$. We show a schematic representation of the general network in Fig. 2, which is similar to the self-regulation circuit. The main differences are that: (i) each DNA type can be regulated by others different proteins than the one expressed by the considered gene (cross regulation), and (ii) the protein degradation rate can be a variable function of all proteins types considered.

The structure of this multidimensional network is equivalent to the previous self-regulation case. Each promoter can switch from the inactive states ($DNAi_{\mathrm {off}}$) to the active one ($DNAi_{\mathrm {on}}$) or vice versa with rate constants $k_{\mathrm {on}}^{i}$ and $k_{\mathrm {off}}^{i}$ respectively. The leakage (basal) messenger RNA production from the inactive promoter is conserved at lower rate constant ($k_{\varepsilon }^i$) than its production from the active state ($k_m^i$). Each *i* messenger RNA type is translated into the protein $X_i$ at rate constant $k_x^i$. Both messengers RNA and proteins are degraded with rates $\gamma _m^i$ and $\gamma _x^i({\mathbf {x}})$ respectively.

Note that for this general network the total rate of production of $mRNA_i$, $R_T^i$, can be written as the rate constant production from the active $DNA_i$ state times one input function $c_i({\mathbf {x}})$ describing all possible types of feedback mechanism. However, there are not universal expressions for $c_i({\mathbf {x}})$, due to their dependence on the regulatory mechanism considered (the messenger RNA production can occur from intermediate DNA states between the total activated and the total repressed ones), some examples have been described in Alon (2007) and Pájaro et al. (2017). Without loss of generality, we can construct the input function verifying that its image is a positive interval, $c_i:{{\mathbb {R}}}_{+}^{n} \rightarrow [\varepsilon _i, \ 1]$, where the leakage constant $\varepsilon _i$ is defined as $k_{\varepsilon }^i/k_m^i$ with $k_{\varepsilon }^i$ being the *mRNAi* rate constant from the total repressed $DNA_i$ (the lowest rate of $mRNA_i$ production).

Considering the set of *n* proteins ${\mathbf {X}}=\{X_1, \ldots , X_n\}$, we define the *n*-vector ${\mathbf {x}}=(x_1, \ldots , x_n) \in {\mathbb {R}}_+^n$ as the amount of each protein type. The generalised (*n*-dimensional) PIDE model, proposed in Pájaro et al. (2017), describes the temporal evolution of the joint density distribution function of *n* proteins $p:{{\mathbb {R}}}_{+}\times {{\mathbb {R}}}_{+}^{n} \rightarrow {{\mathbb {R}}}_{+}$:

1.8 $$\begin{aligned} \dfrac{\partial p}{\partial t}(t,{\mathbf {x}})= & {} \sum _{i=1}^{n}\left( \dfrac{\partial }{\partial x_i}\left[ \gamma _x^i({\mathbf {x}}) x_i p({\mathbf {x}})\right] \nonumber \right. \\&\left. +\, k_m^i \int _0^{x_i} \! \omega _i(x_i-y_i) c_i({\mathbf {y}}_i)p(t,{\mathbf {y}}_i) \, \mathrm {d}y_i -k_m^ic_i({\mathbf {x}})p({\mathbf {x}})\right) \end{aligned}$$

where ${\mathbf {y}}_i$ represents the vector state ${\mathbf {x}}$ with its *i*-th position changed to $y_i$, (that is: $({\mathbf {y}}_i)_j=x_j \ \text {if} \ j\ne i$ and $({\mathbf {y}}_i)_j=y_i \ \text {if} \ j=i$), and $\gamma _x^i({\mathbf {x}})$ is the degradation rate function of each protein. The first term in the right-hand side of the equation accounts for protein degradation whereas the integral describes protein production by bursts. The burst size is assumed to follow an exponential distribution, what leads to the conditional probability for protein jumping from a state $y_i$ to a state $x_i$ after a burst be given by:

$$\begin{aligned} \omega _i(x_i - y_i) = \dfrac{1}{b_i} \exp \left( -\dfrac{x_i-y_i}{b_i} \right) \end{aligned}$$

where $b_i=\frac{k_x^{i}}{\gamma _m^{i}}$ are dimensionless frequencies associated to translation which corresponds with the mean protein produced per burst (burst size). The function $c_i({\mathbf {x}})$ ($c_i:{{\mathbb {R}}}_{+}^{n} \rightarrow [\varepsilon _i, \ 1]$) is an input function, which models the regulation mechanism of the network considered.

The stationary solution $P_{\infty }({\mathbf {x}})$ of (1.8) satisfies:

1.9 $$\begin{aligned}&\sum _{i=1}^{n}\left( \dfrac{\partial }{\partial x_i}\left[ \gamma _x^i({\mathbf {x}}) x_i P_{\infty }({\mathbf {x}})\right] \nonumber \right. \\&\quad \left. +\,k_m^i \int _0^{x_i} \! \omega _i(x_i-y_i) c_i({\mathbf {y}}_i)P_{\infty }({\mathbf {y}}_i) \, \mathrm {d}y_i -k_m^ic_i({\mathbf {x}})P_{\infty }({\mathbf {x}})\right) =0. \end{aligned}$$

Note that an analytical expression for the steady state solution is not known for the general case of the PIDE model (1.8). Some properties of the 1D solution remain valid for the nD steady state since $P_{\infty }({\mathbf {x}})$ is a probability density function, then $\int _{{\mathbb {R}}_+^n}P_{\infty }({\mathbf {x}})\, \mathrm {d} {\mathbf {x}} = 1$. However, we do not have any other prior information about the properties of stationary solutions.

### Main results

In this work we will apply entropy methods in order to analyse the asymptotic equilibration for the kinetic equations (1.3) and (1.8). These equations bear a similar structure to the self-similar fragmentation and the growth-fragmentation equations (Perthame and Ryzhik 2005; Laurençot and Perthame 2009; Doumic 2010; Cáceres et al. 2011; Balagué et al. 2013), used for instance in cell division modelling. In those cases, the transport term makes the cluster size of particles grow while the integral term breaks the particles into pieces of smaller size. In our present models, the transport term degrades the number density of proteins while the integral term makes the protein number density to grow.

In fact, the kinetic equations (1.3) and (1.8) have the structure of linear population models as in Michel et al. (2004, 2005) and Carrillo et al. (2011) for which the so-called general relative entropy applies. This fact already reported in Pájaro et al. (2016) implies the existence of infinitely many Lyapunov functionals for these models useful for different purposes among which to analyse their asymptotic behavior. We will make a summary of the main properties of Eq. (1.3) in Sect. 2 together with a quick treatment of the well-posedness theory for these models. They are easily generalisable to the multidimensional case (1.8).

In Sects. 3 and 4, we will improve over the direct application of the general relative entropy method in Pájaro et al. (2016). On one hand, we study in Sect. 3 the case of gene circuits involving one gene, Eq. (1.3), a direct functional inequality between the $L^2$-relative entropy and its production leading to exponential convergence. In order to fix our setting, we recall that $\omega$ is given by (1.2) for some $b > 0$, and $c = c(x)$ is given by (1.4), for some constants $K > 0$, $H \in {\mathbb {Z}}{\setminus }\{0\}$ and $0 < \epsilon \le 1$; and $a > 0$ is a constant. For $1 \le p < +\infty$ we denote by $L^p(\Omega )$ the usual Lebesgue spaces of real functions *f* on $\Omega$ such that $|f|^p$ is integrable in the Lebesgue sense. We also write $L^p(\Omega , w)$ to denote the corresponding spaces of functions *f* such that $|f|^p$ is integrable with a weight *w*.

#### Theorem 1.1

(Long-time behaviour for the 1-dimensional model) Let $p_0$ be a probability distribution such that $p_0 \in L^1((0,+\infty )) \cap L^2((0,+\infty ), P_\infty ^{-1})$, and let *p* be the mild solution to Eq. (1.3) with initial data $p_0$ (see Definition 2.1). There exists a constant $\lambda > 0$ depending only on the parameters of the equation (and not on $p_0$) such that

$$\begin{aligned} \Vert p(t,\cdot ) - P_\infty \Vert _{L^2((0,+\infty ), P_\infty ^{-1})} \le e^{-\lambda t} \Vert p_0 - P_\infty \Vert _{L^2((0,+\infty ), P_\infty ^{-1})}. \end{aligned}$$

The value of $\lambda$ can be estimated explicitly from the arguments in the proof, though we do not consider the specific value to be a good approximation of the optimal decay rate. The behaviour of the stationary solutions $P_\infty (x)$ near the origin and infinity is crucial for direct functional inequalities involving the relative entropy and its production in the one dimensional case.

What we are showing is essentially a spectral gap in a weighted $L^2$ norm, and some remarks are in order regarding the specific choice of space $L^2((0,+\infty ), P_\infty ^{-1})$ that we have made. As will be seen later, this space is very natural for the technique we are going to use, since the evolution operator is contractive in this norm, and a similar observation is true for any Markov semigroup with an equilibrium. However, it is very likely that this operator also has a spectral gap in $L^2$ norms with different weights, in weighted $L^1$ norms, and in other metrics, as is often the case with Markov operators. In many examples (such as the Fokker-Planck equation) it is known that the spectral gap property breaks for weights which are slowly decaying, so that there may not be a spectral gap in $L^1$, for example. In those cases there are well-known examples of initial data with slowly-decaying tails whose associated solution converges to equilibrium as slowly as one wishes. The same happens for example to the Boltzmann equation from kinetic theory; we refer to Gualdani et al. (2010) for details on the extension of spectral gaps to different weights. So the weight is not only a technical assumption: there may be norms and weights in which the convergence is *not* exponential. However, exponential weights as the ones we use are probably far from being the optimal ones where one can show a similar result.

Section 4 is devoted to the analysis of the multidimensional Eq. (1.8) corresponding to multiple genes involved in the gene transcription. In this case, solutions to the stationary problem (1.9) are not explicit and hence we are not able to control precisely the behaviour of the stationary solutions near the origin and infinity as before. For this reason, we are only able to show convergence towards a unique equilibrium solution assuming its existence with suitable behavior near the origin and infinity:

#### Theorem 1.2

(Long-time behaviour for the *nD* model) Given any mild solution *p* with normalised nonnegative initial data $p_0 \in L^1({\mathbb {R}}_+)$ to Eq. (1.8) and given a normalised stationary solution $P_\infty ({\mathbf {x}})$ to (1.8) satisfying the technical Assumption 4.1 from Sect. 4, it holds that

$$\begin{aligned} \lim _{t \rightarrow \infty } \int _{{{\mathbb {R}}}_{+}^{n}}|p(t,{\mathbf {x}})-P_\infty ({\mathbf {x}})|^2 \mathrm {d} {\mathbf {x}} = 0. \end{aligned}$$

As a consequence, if a normalised stationary solution $P_\infty ({\mathbf {x}})$ of (1.8) and satisfying Assumption 4.1 exists, it is unique.

The proof is based on a weaker variant of our one-dimensional inequality, in which the control between the relative entropy and its production is obtained except for an error term which happens to be small under the assumptions of the behavior of the stationary solution $P_{\infty }({\mathbf {x}})$. Both results of equilibration are illustrated with numerical simulations in their corresponding sections.

## Mathematical preliminaries and entropy methods

### Properties of stationary solutions

Let us start by discussing the basic properties of the one dimensional stationary states to (1.3). The behaviour of the stationary state at zero and at $+\infty$ depends on both $r=a\varepsilon -1$ and *a* due to the presence of the function $\rho (x)$ and its dependence on *H*. It is as follows:

1. If $H>0$, then $P_{\infty }(x) \simeq x^{a-1}$ as $x\rightarrow 0^+$ and $P_{\infty }(x) \simeq x^r e^{-x/b}$ as $x\rightarrow +\infty$. Then the stationary state $P_\infty (x)$ exhibits a singularity at zero for $0<a<1$ and it is smooth otherwise having zero limit for $a> 1$ and a positive limit for $a=1$.
2. If $H< 0$, then $P_{\infty }(x) \simeq x^r$ as $x\rightarrow 0^+$ and $P_{\infty }(x) \simeq x^{a-1} e^{-x/b}$ as $x\rightarrow +\infty$. Then the stationary state $P_\infty (x)$ exhibits a singularity at zero for $a\varepsilon <1$ and it is smooth otherwise having zero limit for $a\varepsilon >1$ and a positive limit for $a\varepsilon =1$.

As a particular case, if $c(x) \equiv 1$ then $P_{\infty }(x)$ is given by (1.7) and we have $P_{\infty }(x) \simeq x^{a-1}$ as $x\rightarrow 0^+$ and $P_{\infty }(x) \simeq x^{a-1} e^{-x/b}$ as $x\rightarrow +\infty$. Then the stationary state $P_\infty (x)$ exhibits a singularity at zero for $a<1$ and it is smooth otherwise having zero limit for $a>1$ and a positive limit for $a=1$.

**Fig. 3:**
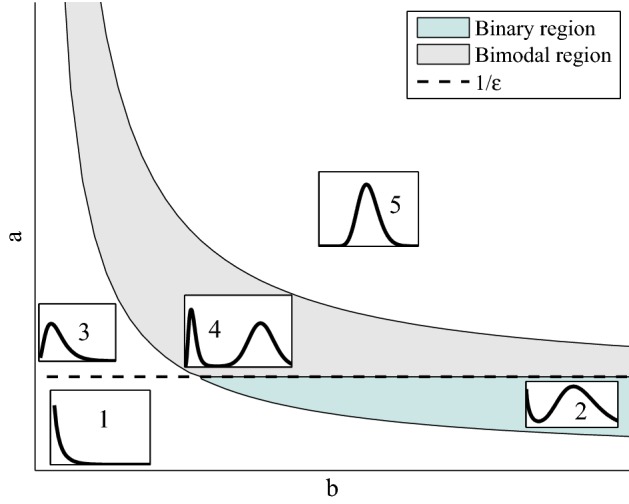
Regions in the parameter space, where protein distribution exhibits different behaviours for $H<0$. There are two large areas where the protein distribution change fundamentally its properties, the first including the shapes one and two, where $a<\frac{1}{\varepsilon }$ and $\lim _{x \rightarrow 0}P_{\infty }(x)=+\infty$ and the second with $P_{\infty }(x)$ finite for all non-negative *x*, which includes shapes three to five

Note that in all cases $\lim _{x \rightarrow \infty } P_{\infty }(x) = 0$. As we can see in Fig 3, the stationary solution has five different qualitative behaviours for $H<0$ (see also Pájaro et al. 2015):

1. If $a<\dfrac{1}{\varepsilon }$, then $\lim _{x \rightarrow 0} P_{\infty }(x) = \infty$. 1.1 Only one peak in $x=0$ (Case 1 Fig  3). 1.2 Two peaks one in $x=0$ and another in $x>0$ (Case 2 Fig  3).
2. If $a > \dfrac{1}{\varepsilon }$, then $\lim _{x \rightarrow 0} P_{\infty }(x) = 0$. If $a \ge \dfrac{1}{\varepsilon }$, then $\lim _{x \rightarrow 0} P_{\infty }(x) = M$ with $M\ge 0$. 2.1 Only one peak in $x > 0$ but close to $x=0$ (Case 3 Fig  3). 2.2 Two different peaks at two points $x_1, x_2 > 0$ (Case 4 Fig 3). 2.3 Only one peak in $x \ge 0$ (Case 5 Fig  3).

Note that, cases 2.1 and 2.3 are equivalent, and $\lim _{x \rightarrow \infty } P_{\infty }(x) = 0$ for all cases. If $H>0$ (or $c(x)=1$) the bimodal behaviour disappears, and only cases 3 or 5 remain for $a>1$ and case 1 if $a<1$.

### Well-posedness

The 1D Eq. (1.3) is a linear integro-differential equation for which well-posedness and some basic properties follow from standard methods. A *classical solution* to Eq. (1.3) with initial data $p_0 \in {\mathcal {C}}^1([0,+\infty ))$ is a function $p \in {\mathcal {C}}^1([0,+\infty ) \times (0,+\infty ))$ which satisfies (1.3) for all $(t,x) \in [0,+\infty ) \times (0,+\infty )$, and such that $p(0,x) = p_0(x)$ for all $x \in (0,+\infty )$. It is not hard to show that, given an integrable initial condition $p_0 \in {\mathcal {C}}^{1,\mathrm {b}}([0,+\infty ))$, there exists a unique mass-conserving classical solution. In order to give a brief sketch of the proof it is perhaps easier to work with *mild solutions*, which we will introduce now. Given $p(t)=p(t,\cdot ) \in L^1(0,+\infty )$, we denote by *L*[*p*(*t*)] the right-hand side of (1.3) given by

$$\begin{aligned} L[p(t)](x) := a \int _0^x \! \omega (x-y)c(y)p(t,y) \, \mathrm {d}y - ac(x)p(t,x), \qquad x > 0, \end{aligned}$$

and given any function $p_0 \, [0,+\infty ) \times (0,+\infty ) \rightarrow {\mathbb {R}}$ we define

$$\begin{aligned} (X_t \# p_0) (x) := p_0(x e^t) e^t, \qquad \text {for}~ t \ge 0, x > 0. \end{aligned}$$

This notation is motivated by the fact that $X_t \# p_0$ is the transport of the function $p_0$ by the dilation map $X_t(x) := x e^{-t}$. By the method of characteristics one easily sees that a classical solution *p* to (1.3) must satisfy

2.1 $$\begin{aligned} p(t,x) = (X_t \# p_0)(x) + \int _0^t \big (X_{t-s} \# L[p(s,\cdot )] \big )(x) \,\mathrm {d}s \qquad \text {for all}~ t \ge 0, x > 0. \end{aligned}$$

This suggests the following definition.

#### Definition 2.1

Let $p_0 \in L^1(0,+\infty )$. We say that $p \in {\mathcal {C}}([0,\infty ); L^1(0,+\infty ))$ is a *mild solution* to Eq. (1.3) with initial data $p_0$ if it satisfies (2.1) for all $t \ge 0$, for almost all $x > 0$.

#### Theorem 2.2

For any $p_0 \in L^1(0,+\infty )$ there exists a unique mild solution of (1.3) with initial data $p_0$ satisfying

$$\begin{aligned} \int _0^\infty p(t,x)\, \mathrm {d}x = \int _0^\infty p_0(x)\, \mathrm {d}x \qquad \text {for all}\,{ t \ge 0.} \end{aligned}$$

In addition, there is a constant $C > 0$ (independent of $p_0$) such that

2.2 $$\begin{aligned} \Vert p(t) \Vert _1 \le e^{Ct} \Vert p_0\Vert _1 \qquad \text {for all}~t \ge 0. \end{aligned}$$

Moreover, for any $p_0 \in {\mathcal {C}}^{1,\mathrm {b}}(0,+\infty )$ there exists a unique classical solution of (1.3) with initial data $p_0$.

#### Proof

This result can be obtained by considering the functional:

$$\begin{aligned} \Phi [p](t,x) := (X_t \# p_0)(x) + \int _0^t \big (X_{t-s} \# L[p(s,\cdot )] \big )(x) \,\mathrm {d}s, \end{aligned}$$

defined on the Banach space

$$\begin{aligned} Y := \{ p \in {\mathcal {C}}([0,T]; L^1(0,+\infty )) \mid p(0) = p_0 \} \end{aligned}$$

with norm

$$\begin{aligned} \Vert p \Vert _Y := \sup _{t \in [0,T]} \Vert p_t \Vert _1, \end{aligned}$$

for $T > 0$ small enough. Note that

$$\begin{aligned} \int _0^\infty \Phi [p](t,x)\, \mathrm {d}x = \int _0^\infty p(t,x)\, \mathrm {d}x = \int _0^\infty p_0(x)\, \mathrm {d}x \qquad \text {for all}~ t \ge 0. \end{aligned}$$

By following an argument very similar to that of Picard iterations, one obtains the existence of mild solutions on a time interval [0, *T*]. Since the equation is linear (and our equation is invariant under time translations), this argument can be iterated to find solutions on $[0,+\infty )$. We refer to Engel and Nagel (2006) and Cañizo et al. (2013) for full details of this standard argument.

If the initial condition $p_0$ is in ${\mathcal {C}}^{1, \mathrm {b}}(0,+\infty )$, one can see that the iteration above can also be done in the space $Z := \{ p \in {\mathcal {C}}^{1,\mathrm {b}}([0,T] \times (0,+\infty )) \mid p(0,x) = p_0(x) \text { for x > 0} \}$. This gives the existence of a unique classical solution in this space. $\square$

The constructed solutions have basic properties: positivity preserving, $L^1$-contraction, and maximum principle.

#### Lemma 2.3

Take $p_0 \in L^1(0,+\infty )$ and let *p* be the unique mild solution to Eq. (1.3) given by Theorem  2.2.

1. Positivity is preserved: if $p_0 \ge 0$ a.e. then $p(t) \ge 0$ a.e., for all $t \ge 0$.
2. The $L^1$ norm is decreasing
  $$\begin{aligned} \Vert p(t) \Vert _1 \le \Vert p_0\Vert _1 \qquad \text {for all}~ {t \ge 0,} \end{aligned}$$
  leading to $L^1$-contraction by linearity. If $p_0 \ge 0$, the above inequality becomes an identity.
3. Maximum principle:
  $$\begin{aligned} \mathop {{{\mathrm{ess\,inf}}}}\limits _{x> 0} \frac{p_0(x)}{P_\infty (x)} \le \frac{p(t,x)}{P_\infty (x)} \le \mathop {{{\mathrm{ess\,sup}}}}\limits _{x > 0} \frac{p_0(x)}{P_\infty (x)}. \end{aligned}$$

#### Proof

In order to show that positivity is preserved for any classical solution, we can rewrite, using Duhamel’s formula,

$$\begin{aligned} p(t) = S_t p_0 + \int _0^t S_{t-s} L^+[p(s)] \,\,\mathrm {d}s =: \Psi (p)(t), \end{aligned}$$

where $S_t$ is the semigroup associated to the equation $\partial _t p - \partial _x (x p) + a c(x) p = 0$ and $L^+$ is the operator given by

$$\begin{aligned} L^+[p(t)](x) := a \int _0^x \omega (x-y)c(y)p(t,y) \,\,\mathrm {d}y \qquad x > 0. \end{aligned}$$

This way of writing the solution clearly shows *p* is nonnegative if $p_0$ is nonnegative, since *p* is a fixed point of the positivity-preserving operator $\Psi$, which is also contractive in the $L^\infty$ norm (for example) for *t* small enough. Now, for a mild solution we obtain the same result by approximation from classical solutions, taking into account the $L^1$-stability (2.2).

For the second part of the result, denote by $T_t$ the semigroup in $L^1(0,+\infty )$ defined by the equation, and write $f_+ := \max \{0, f\}$, $f_- := \max \{0, -f\}$ for the positive and negative parts of a function *f*, so that $f = f_+ - f_-$. The positivity and mass preservation imply that:

$$\begin{aligned} \Vert p(t) \Vert _1= & {} \Vert T_t p_0 \Vert _1 \le \Vert T_t ((p_0)_+) \Vert _1 + \Vert T_t ((p_0)_-) \Vert _1 \\= & {} \int T_t ((p_0)_+) + \int T_t ((p_0)_-) = \int (p_0)_+ + \int (p_0)_- = \Vert p_0 \Vert _1. \end{aligned}$$

Finally, for the maximum principle just notice that, if *M* is the supremum on the right hand side, the function $q = M P_\infty - p$ is a mild solution with nonnegative initial data. Due to preservation of positivity we obtain the inequality on the right-hand side. The minimum principle is obtained analogously. $\square$

### Entropy and *H*-theorem

Let $H \, [0,+\infty ) \rightarrow {\mathbb {R}}$ be a convex function. We define the general relative entropy functional as:

2.3 $$\begin{aligned} {\mathcal {G}}_H(u)(t)= \int _{0}^{\infty } H(u(t,x)) P_{\infty }(x) \mathrm {d}x, \end{aligned}$$

with $u(t,x):=p(t,x)/P_\infty (x)$. The basic general relative entropy principle is that ${\mathcal {G}}_H(p(t)/P_\infty )$ is a decreasing quantity when *p*(*t*) is a solution to (1.3), see Michel et al. (2004, 2005), Carrillo et al. (2011) and Pájaro et al. (2016).

#### Proposition 2.4

Let $H \, [0,+\infty ) \rightarrow {\mathbb {R}}$ is a convex function in ${\mathcal {C}}^1([0,+\infty ))$ and let *p* be a classical solution to (1.3) with integrable initial condition $p_0 \in {\mathcal {C}}^{1,b}[0,+\infty )$ such that $|p_0(x)| \le M P_\infty (x)$ for some $M > 0$. Thus, the relative entropy satisfies

2.4 $$\begin{aligned} \dfrac{\mathrm {d}{\mathcal {G}}_H(u)}{\mathrm {d} t}= & {} a\!\int _{0}^{\infty }\!\!\!\!\int _{y}^{\infty }\!\!\omega (x-y)\Big ( H(u(x)) - H(u(y))\nonumber \\&+\,H'(u(x))\left( u(y)-u(x)\right) \Big )c(y)P_{\infty }(y) \,\mathrm {d} x \mathrm {d} y\le 0, \end{aligned}$$

for all $$t\ge 0$$.

#### Remark 2.5

Notice that the dependence on the time variable in (2.4) has been omitted for simplicity. Observe that the right-hand side in (2.4) is non-positive since the convexity of *H* implies $H(u)-H(v)+H'(u)(v-u) \le 0$ for all $u,v\in {\mathbb {R}}$.

Proposition 2.4 is very close to the results in Section 2 of Michel et al. (2005), but is strictly not contained there due to the form of the integral operator. It is worth giving a derivation of the result, so we include a proof here. We first obtain a technical lemma involving some classical computations in Michel et al. (2005):

#### Lemma 2.6

Under the assumptions of Proposition  2.4, then the following equality is satisfied

2.5 $$\begin{aligned} H'(u(x))\dfrac{\partial [xp(x)]}{\partial x}= & {} \dfrac{\partial [H(u(x))xP_{\infty }(x)]}{\partial x} \nonumber \\&+ \left[ u(x)H'(u(x))-H(u(x))\right] \dfrac{\partial [xP_{\infty }(x)]}{\partial x}. \end{aligned}$$

#### Proof

We know that

$$\begin{aligned} \dfrac{\partial H(u(x))}{\partial x}=H'(u(x))\dfrac{\partial u}{\partial x}=\dfrac{H'(u(x))}{P_{\infty }(x)}\left( \dfrac{\partial p}{\partial x} -u(x)\dfrac{\partial P_{\infty }}{\partial x} \right) , \end{aligned}$$

and

$$\begin{aligned} \dfrac{\partial [H(u(x))xP_{\infty }(x)]}{\partial x}= xP_{\infty }(x)\dfrac{\partial H(u(x))}{\partial x} +H(u(x))\dfrac{\partial [xP_{\infty }(x)]}{\partial x}. \end{aligned}$$

So that, replacing the first expression in the second we have that:

2.6 $$\begin{aligned} \dfrac{\partial [H(u(x))xP_{\infty }(x)]}{\partial x}= & {} xH'(u(x))\left( \dfrac{\partial p}{\partial x} -u(x)\dfrac{\partial P_{\infty }}{\partial x} \right) \nonumber \\&+H(u(x))\dfrac{\partial [xP_{\infty }(x)]}{\partial x}. \end{aligned}$$

Next, by using the following identities:

$$\begin{aligned} x\dfrac{\partial p}{\partial x}= \dfrac{\partial [x p(x)]}{\partial x}-p(x) ~~\text {and}~~ x\dfrac{\partial P_{\infty }}{\partial x}= \dfrac{\partial [x P_{\infty }(x)]}{\partial x}-P_{\infty }(x), \end{aligned}$$

in (2.6) we obtain:

$$\begin{aligned} \dfrac{\partial [H(u(x))xP_{\infty }(x)]}{\partial x}&= H'(u(x))\left( \dfrac{\partial [x p(x)]}{\partial x} -p(x) -u(x)\left( \dfrac{\partial [xP_{\infty }(x)]}{\partial x} - P_{\infty }(x)\right) \right) \\&\ \quad +H(u(x))\dfrac{\partial [xP_{\infty }(x)]}{\partial x}\\&= H'(u(x))\left( \dfrac{\partial [x p(x)]}{\partial x} -u(x)\dfrac{\partial [xP_{\infty }(x)]}{\partial x} \right) \\&\ \quad +H(u(x))\dfrac{\partial [xP_{\infty }(x)]}{\partial x}. \end{aligned}$$

Note that the terms $u(x)P_{\infty }(x)-p(x)$ vanish, since $u(x)P_{\infty }=p(x)$. Finally, reordering terms in the last equation we obtain the equality (2.5). $\square$

#### Proof of Proposition  2.4

We start the proof computing the time derivative of the general relative entropy functional

$$\begin{aligned} \dfrac{\mathrm {d}{\mathcal {G}}_H(u)}{\mathrm {d}t}&= \dfrac{\partial }{\partial t} \int _{0}^{\infty }H(u(x))P_{\infty }(x)\mathrm {d}x\\&=\int _{0}^{\infty }\dfrac{\partial }{\partial t} H(u(x))P_{\infty }(x)\mathrm {d}x=\int _{0}^{\infty }H'(u(x))\dfrac{\partial p}{\partial t} \mathrm {d}x . \end{aligned}$$

We replace the time derivative of $p(\tau ,x)$ by its expression (1.3) to obtain:

$$\begin{aligned} \dfrac{\mathrm {d}{\mathcal {G}}_H(u)}{\mathrm {d}t}= \int _{0}^{\infty }H'(u(x))\left( \dfrac{\partial [xp(x)]}{\partial x} + a \int _0^x \omega (x-y)c(y)p(y) \mathrm {d}y - ac(x)p(x) \right) \mathrm {d}x . \end{aligned}$$

Using lemma 2.6 and the fact that $p(x)=u(x)P_{\infty }(x)$ we have:

$$\begin{aligned} \dfrac{\mathrm {d}{\mathcal {G}}_H(u)}{\mathrm {d}t}=&\, \int _{0}^{\infty }\left( \dfrac{\partial [H(u(x))xP_{\infty }(x)]}{\partial x} + \left( u(x)H'(u(x))-H(u(x))\right) \dfrac{\partial [xP_{\infty }(x)]}{\partial x}\right) \mathrm {d}x \\&\, +a\int _{0}^{\infty }H'(u(x))\left( \int _0^x \omega (x-y)c(y)u(y)P_{\infty }(y) \mathrm {d}y - c(x)u(x)P_{\infty }(x)\right) \mathrm {d}x . \end{aligned}$$

In the above equation the term

$$\begin{aligned} \int _{0}^{\infty }\frac{\partial [H(u(x))xP_{\infty }(x)]}{\partial x}\mathrm {d}x \end{aligned}$$

vanishes since $\lim _{x \rightarrow +\infty } x P_\infty (x) = \lim _{x \rightarrow 0} x P_\infty (x) = 0$, and noticing that $u(x) \le M$ for all $t\ge 0$, $x > 0$ due to the maximum principle in Lemma  2.3. Replacing the term containing the first order derivative by its value in Eq. (1.5) we get

$$\begin{aligned} \dfrac{\mathrm {d}{\mathcal {G}}_H(u)}{\mathrm {d}t}&= -a\int _{0}^{\infty }\left( u(x)H'(u(x))-H(u(x))\right) \\&\quad \ \times \left( \int _0^x \omega (x-y)c(y)P_{\infty }(y) \mathrm {d}y - c(x)P_{\infty }(x)\right) \mathrm {d}x \\&\quad \ +a\int _{0}^{\infty }H'(u(x))\\&\quad \ \times \left( \int _0^x \omega (x-y)c(y)u(y)P_{\infty }(y) \mathrm {d}y - c(x)u(x)P_{\infty }(x)\right) \mathrm {d}x . \end{aligned}$$

Reordering terms in the above equation we have that

$$\begin{aligned} \dfrac{\mathrm {d}{\mathcal {G}}_H(u)}{\mathrm {d}t}&= a\int _{0}^{\infty }H(u(x))\\&\quad \ \times \left( \int _0^x \omega (x-y)c(y)P_{\infty }(y) \mathrm {d}y - c(x)P_{\infty }(x)\right) \mathrm {d}x \\&\quad \ +a\int _{0}^{\infty }H'(u(x))\\&\quad \ \times \left( \int _0^x \omega (x-y)c(y)u(y)P_{\infty }(y) \mathrm {d}y -u(x)\int _0^x \omega (x-y)c(y)P_{\infty }(y) \mathrm {d}y \right) \mathrm {d}x . \end{aligned}$$

Note that

$$\begin{aligned} \int _{0}^{\infty }H(u(x)) c(x)P_{\infty }(x)\mathrm {d}x=\int _{0}^{\infty }H(u(y)) c(y)P_{\infty }(y)\mathrm {d}y, \end{aligned}$$

so we can change the order of integration in the above equation to obtain

$$\begin{aligned} \dfrac{\mathrm {d}{\mathcal {G}}_H(u)}{\mathrm {d}t}=&\, a\int _{0}^{\infty }\left( \int _y^{\infty } \omega (x-y)H(u(x)) \mathrm {d}x c(y)P_{\infty }(y) - H(u(y))c(y)P_{\infty }(y)\right) \mathrm {d}y \\&\, +a\int _{0}^{\infty } \int _y^{\infty } \omega (x-y)\left[ H'(u(x))\left( u(y)-u(x)\right) \right] c(y)P_{\infty }(y) \mathrm {d} x \mathrm {d}y . \end{aligned}$$

Since $\int _{y}^{\infty }\omega (x-y)\mathrm {d}x=1$, we multiply by this integral the second term in the first line on the right-hand side of the above equation to conclude

$$\begin{aligned} \dfrac{\mathrm {d}{\mathcal {G}}_H(u)}{\mathrm {d}t}=&\, a\int _{0}^{\infty } \int _y^{\infty } \omega (x-y) \left[ H(u(x)) - H(u(y))\right] c(y)P_{\infty }(y)\mathrm {d}x \mathrm {d}y \\&\, +a\int _{0}^{\infty } \int _y^{\infty } \omega (x-y)\left[ H'(u(x))\left( u(y)-u(x)\right) \right] c(y)P_{\infty }(y) \mathrm {d} x \mathrm {d}y , \end{aligned}$$

which is the desired identity. $\square$

## Exponential convergence for the 1D PIDE model

In this section our aim is to prove that Eq. (1.3) converges exponentially to the steady state, $P_{\infty }$. For this purpose, we consider the $L^2$-relative entropy, i.e., the convex function *H* is chosen as $H(u)=(u-1)^2$, and

$$\begin{aligned} {\mathcal {G}}_2(u)(t):&=\int _{0}^{\infty }P_\infty (x)(u(t,x)-1)^2 \mathrm {d}x\\&= \int _{0}^{\infty }\dfrac{p^2(t,x)}{P_\infty ^2(x)}P_\infty (x) \mathrm {d}x -1 = \int _{0}^{\infty } u^2(t,x) P_\infty (x) \mathrm {d}x -1, \end{aligned}$$

where we have used that *p*(*t*, *x*) and $P_\infty (x)$ are probability density functions. Now, by replacing the value of the considered convex function in Proposition 2.4, we obtain the following identity

3.1 $$\begin{aligned} {\mathcal {D}}_2(u)(t):= & {} -\dfrac{\mathrm {d}{\mathcal {G}}_2(u)}{\mathrm {d} t}\nonumber \\= & {} a\int _{0}^{\infty }\int _{y}^{\infty }\omega (x-y)\left( u(t,x) - u(t,y) \right) ^2 c(y)P_\infty (y) \mathrm {d} x \mathrm {d} y. \end{aligned}$$

The entropy method consists in finding conditions under which the following functional inequality holds:

3.2 $$\begin{aligned} {\mathcal {G}}_2(u) \le \dfrac{1}{2\beta }{\mathcal {D}}_2(u). \end{aligned}$$

Notice that the dependence on the time variable can be forgotten at this point, since our objective is to show such an inequality among a subset of suitable probability densities. For this purpose, we start by rewriting ${\mathcal {G}}_2(u)$ in a equivalent form (Cáceres et al. 2011):

### Lemma 3.1

Given a non-negative measurable function $P_{\infty } : (0, \ \infty )\rightarrow {{\mathbb {R}}}_{+}$ such that $\int _{0}^{\infty }P_{\infty }(x)\mathrm {d} x =1$ and defining the functional

$$\begin{aligned} {\mathcal {H}}_2(u):=\int _{0}^{\infty }\int _{y}^{\infty }P_{\infty }(x)P_{\infty }(y)\left( u(x) - u(y) \right) ^2 \mathrm {d} x \mathrm {d} y , \end{aligned}$$

there holds $${\mathcal {G}}_2(u)={\mathcal {H}}_2(u)$$.

### Proof

Expanding the square implies

3.3 $$\begin{aligned} {\mathcal {G}}_2(u)=\int _{0}^{\infty }P_{\infty }(x)(u(x)-1)^2 \mathrm {d}x= \int _{0}^{\infty }P_{\infty }(x)u(x)^2 \mathrm {d}x -1, \end{aligned}$$

while ${\mathcal {H}}_2(u)$ is a symmetric function, so that:

$$\begin{aligned} {\mathcal {H}}_2(u)(\tau ) =&\, \dfrac{1}{2}\int _{0}^{\infty }\int _{0}^{\infty }P_{\infty }(x)P_{\infty }(y)\left( u(x) - u(y) \right) ^2 \mathrm {d} x \mathrm {d} y \\ =&\, \dfrac{1}{2}\int _{0}^{\infty }\int _{0}^{\infty }P_{\infty }(x)P_{\infty }(y)\left( u(x)^2 -2u(x) u(y) +u(y)^2 \right) \mathrm {d} x \mathrm {d} y \\ =&\, \int _{0}^{\infty }\int _{0}^{\infty }P_{\infty }(x)P_{\infty }(y)u(x)^2 \mathrm {d} x \mathrm {d} y \\&\quad - \int _{0}^{\infty }\int _{0}^{\infty }P_{\infty }(x)P_{\infty }(y)u(x) u(y) \mathrm {d} x \mathrm {d} y \\ =&\, \int _{0}^{\infty } P_{\infty }(x)u(x)^2 \left( \int _{0}^{\infty }P_{\infty }(y) \mathrm {d} y\right) \mathrm {d} x - \int _{0}^{\infty }\int _{0}^{\infty }p(x) p(y) \mathrm {d} x \mathrm {d} y\\ =&\, \int _{0}^{\infty }P_{\infty }(x)u(x)^2 \mathrm {d}x -1, \end{aligned}$$

which is equal to (3.3). $\square$

As consequence of this lemma we are reduced to show the inequality

3.4 $$\begin{aligned} {\mathcal {H}}_2(u) \le \dfrac{1}{2\beta }{\mathcal {D}}_2(u), \end{aligned}$$

among a suitable subset of probability densities.

### Entropy-entropy production inequality

We start by obtaining bounds for the steady state solution $P_{\infty }$, of the Friedman Eq. (1.3).

#### Lemma 3.2

($P_{\infty }$bounds) For $\delta >0$ we define the intervals of length $\frac{1}{2}$:

$$\begin{aligned} I_{k,\delta }:=\left( \delta +\frac{k}{2}, \ \delta +\frac{k+1}{2} \right] , \qquad k\ge 0 ~~\text {integer}, \end{aligned}$$

and

$$\begin{aligned} p_k:= C\left[ \left( \delta +\frac{k}{2}\right) ^H +K^H \right] ^{\frac{a(\varepsilon -1)}{H}}\left( \delta +\frac{k}{2}\right) ^{a-1}e^{\frac{-(\delta +\frac{k}{2})}{b}}=P_{\infty }\left( \delta +\frac{k}{2}\right) . \end{aligned}$$

Then, the following inequality holds:

3.5 $$\begin{aligned} A(\delta )\le \dfrac{P_{\infty }(x)}{p_k} \le B(\delta ), \qquad \forall x \in I_{k,\delta } ~~\text {and}~~ \forall k, \end{aligned}$$

with $P_{\infty }(x)$ given by (1.6) and $A(\delta )$ and $B(\delta )$ being positive constants that only depend on $\delta$ (and network parameters), but they are independent of protein amount *k*.

#### Proof

Note that $\left[ x^H +K^H \right] ^{\frac{a(\varepsilon -1)}{H}}$ and $e^{\frac{-x}{b}}$ are decreasing functions, so that their maxima are at ${\bar{x}}_0=\delta +\frac{k}{2}$ and their minima are at ${\bar{x}}_1=\delta +\frac{k+1}{2}$ in $I_{k,\delta }$. The term $x^{a-1}$ shows different behaviours which depend on the parameter *a*, (this term is increasing if $a>1$, constant if $a=1$ and decreasing if $a<1$). So that, we can bound $P_{\infty }(x)$ in the interval $I_{k,\delta }$ as follows:

3.6 $$\begin{aligned} \left\{ \begin{array}{ll} g({\bar{x}}_1)(\delta +\frac{k}{2})^{a-1} \le P_{\infty }(x) \le g({\bar{x}}_0)\left( \delta +\frac{k+1}{2}\right) ^{a-1} &{} \qquad \text {if}~~ a>1 \\ &{}\\ g({\bar{x}}_1) \le P_{\infty }(x) \le g({\bar{x}}_0) &{} \qquad \text {if}~~ a=1 \\ &{}\\ g({\bar{x}}_1)\left( \delta +\frac{k+1}{2}\right) ^{a-1} \le P_{\infty }(x) \le g({\bar{x}}_0)\left( \delta +\frac{k}{2}\right) ^{a-1} &{}\qquad \text {if}~~ a<1 \end{array} \right. \end{aligned}$$

where $$g(x)=Z\left[ x^H +K^H \right] ^{\frac{a(\varepsilon -1)}{H}}e^{\frac{-x}{b}}$$.

Now, in order to calculate the bounds of $\frac{P_{\infty }(x)}{p_k}$, we divide the expression (3.6) by $p_k$ to obtain $A(\delta ,k) \le \frac{P_{\infty }(x)}{p_k} \le B(\delta ,k)$ with the functions *A* and *B* being,

$$\begin{aligned} A(\delta ,k):=\left\{ \begin{array}{lr} \left( \dfrac{(\delta +\frac{k+1}{2})^H +K^H}{(\delta +\frac{k}{2})^H +K^H}\right) ^{\frac{a(\varepsilon -1)}{H}}e^{\frac{-1}{2b}} &{} \qquad \text {if}~~ a\ge 1\\ &{}\\ \left( \dfrac{(\delta +\frac{k+1}{2})^H +K^H}{(\delta +\frac{k}{2})^H +K^H}\right) ^{\frac{a(\varepsilon -1)}{H}}e^{\frac{-1}{2b}}\left( \dfrac{2\delta +k+1}{2\delta +k}\right) ^{a-1} &{} \qquad \text {if}~~ a<1 \end{array}\right. \end{aligned}$$

and

$$\begin{aligned} B(\delta ,k):=\left\{ \begin{array}{lr} \left( \dfrac{2\delta +k+1}{2\delta +k}\right) ^{a-1} &{} \qquad \text {if}~~ a > 1\\ &{}\\ 1 &{} \qquad \text {if}~~ a \le 1 \end{array}\right. \end{aligned}$$

Notice that,

$$\begin{aligned} \lim _{k \rightarrow \infty } A(\delta ,k)=e^{-\frac{1}{2b}}, \qquad \lim _{k \rightarrow \infty } B(\delta ,k)=1, \end{aligned}$$

implies that $A(\delta ):=\underset{k\ge 0}{\min }\left( A(\delta ,k)\right)$ and $B(\delta ):=\underset{k\ge 0}{\max }\left( B(\delta ,k)\right)$ are well-defined and positive, leading to desired inequality (3.5). $\square$

Note that inequality (3.5) can be directly checked for the simplest open loop case, whose stationary solution is given by (1.7).

#### Lemma 3.3

Let us define

3.7 $$\begin{aligned} M_j:=\sum _{k=1}^{j-1}\dfrac{1}{m_k}, \end{aligned}$$

with $\{m_k\}_{k\ge 1}$ a positive sequence given by $m_k= p_k e^{\frac{\delta +\frac{k}{2}}{2b}}$. Then, there exists $C>0$ such that

3.8 $$\begin{aligned} m_k \sum _{j=k+1}^{\infty } M_j p_j \le C p_k ,\qquad \text{ for } \text{ all } k\in {\mathbb {N}}. \end{aligned}$$

#### Proof

We define $\{a_j\}_{j\ge 1}$ with $a_j=\frac{1}{m_j}$ to calculate the following limit

$$\begin{aligned} \lim _{j \rightarrow \infty } \dfrac{a_{j+1}-a_j}{M_{j+1}-M_j}= & {} \lim _{j \rightarrow \infty } \left( \dfrac{\left( \left( \delta + \frac{j+1}{2}\right) ^H +K^H\right) ^{\frac{a(1-\varepsilon )}{H}}\left( \delta +\frac{j+1}{2}\right) ^{1-a}}{\left( \left( \delta + \frac{j}{2}\right) ^H +K^H\right) ^{\frac{a(1-\varepsilon )}{H}}\left( \delta +\frac{j}{2}\right) ^{1-a}}e^{\frac{1}{4b}} -1\right) \\= & {} e^{\frac{1}{4b}} -1. \end{aligned}$$

Since this limit exists and $\{M_j\}_{j\ge 1}$ is a strictly increasing and divergent sequence, we can use the Stolz-Cesàro theorem to obtain that $M_j\le C_0 a_j$, with $C_0>0$ constant. Then,

$$\begin{aligned} m_k \sum _{j=k+1}^{\infty } M_j p_j \le C_{0}m_k \sum _{j=k+1}^{\infty } a_j p_j. \end{aligned}$$

The summation term at the right hand side can be calculated as follows

$$\begin{aligned} \sum _{j=k+1}^{\infty } a_j p_j= \sum _{j=k+1}^{\infty } e^{-\frac{2\delta +j}{4b}} =\dfrac{e^{-\frac{2b-1}{4b}}}{e-1}e^{-\frac{2\delta +k}{4b}}, \end{aligned}$$

so that

$$\begin{aligned} m_k \sum _{j=k+1}^{\infty } M_j p_j \le C m_k e^{-\frac{2\delta +k}{4b}}= C p_k, \end{aligned}$$

with $C=C_0 \frac{e^{-\frac{2b-1}{4b}}}{e-1}$, concluding the proof. $\square$

In order to prove the exponential convergence of the Friedman Eq. (1.3) we are going to split the proof of inequality (3.4) in the following two propositions.

#### Proposition 3.4

There exists $\lambda >0$ such that

3.9 $$\begin{aligned} \lambda {\mathcal {H}}_2(u) \le \int _{0}^{\infty }\int _{y}^{y+1}P_{\infty }(y)\left( u(x) - u(y) \right) ^2 \mathrm {d} x \mathrm {d} y :=D(u), \end{aligned}$$

with $u=p/P_\infty$, for all $p \in L^1((0,+\infty )) \cap L^2((0,+\infty ), P_\infty ^{-1})$.

#### Proof

We take $0<\delta <1$ and split ${\mathcal {H}}_2(u)$ in two parts

$$\begin{aligned} {\mathcal {H}}_2(u)=&\int _{\delta }^{\infty }\int _{y}^{\infty }P_{\infty }(x)P_{\infty }(y)\left( u(x) - u(y) \right) ^2 \mathrm {d} x \mathrm {d} y \\&+\int _{0}^{\delta }\int _{y}^{\infty }P_{\infty }(x)P_{\infty }(y)\left( u(x) - u(y) \right) ^2 \mathrm {d} x \mathrm {d} y := {\mathcal {H}}_{21}(u)+{\mathcal {H}}_{22}(u). \end{aligned}$$

For $i,j\ge 0$ integers we define

$$\begin{aligned} A_{i,j}:=\int _{I_{i,\delta }}\int _{I_{j,\delta }}\left( u(x)-u(y)\right) ^2 \mathrm {d} y \mathrm {d} x =\int _{I_{i,\delta }}\int _{I_{j,\delta }}\left( u(x)-u(y)\right) ^2 \mathrm {d} x \mathrm {d} y. \end{aligned}$$

We can estimate both the left and the right-hand sides of (3.9) by using the quantities $A_{i,j}$.

**Step 1:**${\mathcal {H}}_{21}(u)$ bound.- We start working on the term ${\mathcal {H}}_{21}(u)(\tau )$, where $0<\delta<y<x$. By swapping (*x*, *y*) in the domain of integration, we get

$$\begin{aligned} {\mathcal {H}}_{21}(u)=&\int _{\delta }^{\infty }\int _{\delta }^{x}P_{\infty }(x)P_{\infty }(y)\left( u(x) - u(y) \right) ^2 \mathrm {d} y \mathrm {d} x \\ \le&\sum _{i=0}^{\infty }\sum _{j=0}^{i}\int _{I_{i,\delta }}\int _{I_{j,\delta }}\left( u(x)-u(y)\right) ^2P_{\infty }(x)P_{\infty }(y) \mathrm {d} y \mathrm {d} x. \end{aligned}$$

Now, using the inequality (3.5) and the symmetry $A_{i,j}=A_{j,i}$, we obtain

3.10 $$\begin{aligned} {\mathcal {H}}_{21}(u)&\le B(\delta ) ^2 \sum _{i=0}^{\infty }\sum _{j=0}^{i} p_i p_j\int _{I_{i,\delta }}\int _{I_{j,\delta }}\!\!\!\!\left( u(x)-u(y)\right) ^2 \mathrm {d} y \mathrm {d} x \nonumber \\&= B(\delta ) ^2 \sum _{i=0}^{\infty }\sum _{j=0}^{i} p_i p_j A_{i,j}\nonumber \\&= B(\delta ) ^2 \sum _{j=0}^{\infty }\sum _{i=j}^{\infty } p_i p_j A_{i,j}= B(\delta ) ^2 \sum _{i=0}^{\infty }\sum _{j=i}^{\infty } p_i p_j A_{i,j}. \end{aligned}$$

Note that some terms in this expression already appear in the right hand side of (3.9), since:

3.11 $$\begin{aligned} \sum _{i=0}^{\infty } p_i^2 A_{i,i}=&\, \sum _{i=0}^{\infty } p_i p_i \int _{I_{i,\delta }}\int _{I_{i,\delta }}\left( u(x)-u(y)\right) ^2 \mathrm {d} x \mathrm {d} y \nonumber \\ \le&\, \dfrac{1}{A(\delta )^2}\sum _{i=0}^{\infty } \int _{I_{i,\delta }}\int _{I_{i,\delta }}\left( u(x)-u(y)\right) ^2 P_{\infty }(x)P_{\infty }(y) \mathrm {d} x \mathrm {d} y \nonumber \\ =&\, \dfrac{2}{A(\delta )^2}\sum _{i=0}^{\infty } \int _{I_{i,\delta }}\int _{\underset{x>y}{x \in I_{i,\delta }}}\left( u(x)-u(y)\right) ^2 P_{\infty }(x)P_{\infty }(y) \mathrm {d} x \mathrm {d} y \nonumber \\ \le&\, \dfrac{2}{A(\delta )^2}\sum _{i=0}^{\infty } \int _{I_{i,\delta }}\int _{y}^{y+1}\left( u(x)-u(y)\right) ^2 P_{\infty }(x)P_{\infty }(y) \mathrm {d} x \mathrm {d} y \nonumber \\ =&\, \dfrac{2}{A(\delta )^2} \int _{\delta }^{\infty }\int _{y}^{y+1}\left( u(x)-u(y)\right) ^2 P_{\infty }(x)P_{\infty }(y) \mathrm {d} x \mathrm {d} y \nonumber \\ \le&\, \dfrac{P_M}{A(\delta )^2} \int _{\delta }^{\infty } \int _{y}^{y+1}\left( u(x)-u(y)\right) ^2 P_{\infty }(y) \mathrm {d} x \mathrm {d} y \le \dfrac{ P_M}{A(\delta )^2} D(u), \end{aligned}$$

where $P_M=\underset{x \in [\delta , \ \infty )}{\max } P_{\infty }(x) < \infty$ due to the properties described in Sect. 2.1.

In order to estimate $A_{i,j}$ for $j>i$ we fix *i*, *j* and call $n:=j-i\ge 1$. We use $n-1$ “intermediate reactions” to write the following: introduce $n-1$ dummy integration variables $z_{i+1}, \dots , z_{j-1}$ and denote averaged integrals with a stroke. Thus, we have:where the last step is just renaming $x\equiv z_j$ and $y\equiv z_i$. Observe that nothing has been done in the case $j=i+1$. Using the Cauchy-Schwarz inequality and (3.7), we haveHence, we deduce that

$$\begin{aligned} A_{i,j} \le M_j\sum _{k=i}^{j-1}m_k A_{k,k+1} \qquad \text {for all}~ {j>i.} \end{aligned}$$

Thus, we get

$$\begin{aligned} \sum _{i=0}^{\infty }\sum _{j=i+1}^{\infty }p_i p_j A_{i,j}&\le \sum _{i=0}^{\infty }\sum _{j=i+1}^{\infty }p_i p_j M_j\sum _{k=i}^{j-1}m_k A_{k,k+1}\\&= \sum _{k=0}^{\infty } m_k A_{k,k+1}\sum _{j=k+1}^{\infty } p_j M_j\sum _{i=0}^{k}p_i \\&\le C_{\delta }^1\sum _{k=0}^{\infty } A_{k,k+1} m_k \sum _{j=k+1}^{\infty } M_j p_j. \end{aligned}$$

The inequality $\sum _{i=0}^{k}p_i\le C$, in the previous expression, holds because $\sum _{i=0}^{\infty }p_i$ is a convergent series due to the d’Alembert’s ratio test. Moreover, (3.8) implies

3.12 $$\begin{aligned} \sum _{i=0}^{\infty }\sum _{j=i+1}^{\infty }p_i p_j A_{i,j} \le C \sum _{k=0}^{\infty } A_{k,k+1} p_k \end{aligned}$$

for a generic constant $C>0$. We finally work in the Eq. (3.12) to obtain

$$\begin{aligned} \sum _{k=0}^{\infty } A_{k,k+1} p_k =&\, \sum _{k=0}^{\infty } \int _{I_{k,\delta }}\int _{I_{k+1,\delta }}\left( u(x)-u(y)\right) ^2 \mathrm {d} x \, p_k \, \mathrm {d} y\\ \le&\, \frac{1}{A(\delta )}\sum _{k=0}^{\infty } \int _{I_{k,\delta }}\int _y^{y+1}\left( u(x)-u(y)\right) ^2 \mathrm {d} x P_{\infty }(y)\mathrm {d} y\\ \le&\, \frac{1}{A(\delta )} \int _0^{\infty }\int _y^{y+1}\left( u(x)-u(y)\right) ^2 P_{\infty }(y) \mathrm {d} x \mathrm {d} y=\frac{1}{A(\delta )} D(u), \end{aligned}$$

where we use that $y<\delta + \frac{k+1}{2}<\delta + \frac{k+2}{2} < y+1$ and (3.5). We conclude by plugging the above estimate in (3.12), which together with Eqs. (3.10) and (3.11) show that

3.13 $$\begin{aligned} \lambda _1{\mathcal {H}}_{21}(u) \le D(u), \end{aligned}$$

for some constant $\lambda _1>0$.

**Step 2:**${\mathcal {H}}_{22}(u)$ bound.- To prove that there exists $\lambda _2>0$ such that

$$\begin{aligned} \lambda _2 {\mathcal {H}}_{22}(u) \le \int _{0}^{\infty }\int _{y}^{y+1}P_{\infty }(y)\left( u(x) - u(y) \right) ^2 \mathrm {d} x \mathrm {d} y , \end{aligned}$$

we use an intermediate variable $z\in (\delta , \ 1)$ as follows:We bound each of the terms $I_1, \ I_2$. First, for $I_1$ we deduce thatsince $\int _{0}^{\infty }P_{\infty }(y)\mathrm {d} y=1$. For $I_{11}$ we use that $P_\infty$ is bounded below on $[\delta , \ 1]$ ($\frac{1}{C_{\delta }}\le P_\infty (x), \ x \in [\delta , \ 1]$) to deduceNote that the right hand side of the above equation is bounded by a multiple of the term ${\mathcal {H}}_{21}(u)$, thus leading to $I_{11}\le C{\mathcal {H}}_{21}(u)$ with $C=\dfrac{2C_{\delta }}{1-\delta }$. Using (3.13) we deduce that $I_{11}\le CD(u)$.

The integral $I_{12}$ is clearly smaller than the right hand side of (3.9) since it involves a smaller domain of integration, indeed we obtainsince $z<\delta<x<1<z+1$. For $I_2(\tau )$, notice thatand thus, we also deduce that $I_2\le CD(u)$. Putting together the estimates on $I_{11}$, $I_{12}$ and $I_2$, we conclude that

3.14 $$\begin{aligned} \lambda _2{\mathcal {H}}_{22}(u) \le D(u), \end{aligned}$$

for some $\lambda _2>0$. Finally, inequalities (3.13) and (3.14) together imply that $\lambda {\mathcal {H}}_{2}(u) \le D(u)$ concluding the proof. $\square$

#### Proposition 3.5

There exists $\alpha >0$ such that

3.15 $$\begin{aligned} \alpha D(u)\le {\mathcal {D}}_2(u). \end{aligned}$$

with $u=p/P_\infty$, for all $p \in L^1((0,+\infty )) \cap L^2((0,+\infty ), P_\infty ^{-1})$.

#### Proof

Note that, $y<x<y+1$ on the left hand side of (3.15). Thus, we can bound the term $\omega (x-y)$ with $x \in [y, \ y+1]$. Since $\omega (x)$ is a decreasing function of *x*, then

$$\begin{aligned} \omega (1)=\frac{1}{b} e ^{\frac{-1}{b}}\le \omega (x-y) \le \frac{1}{b}=\omega (0) \qquad \text {with}~ x \in [y, \ y+1] ~\hbox {and}~ y \in {\mathbb {R}}^+. \end{aligned}$$

Moreover, the term *c*(*x*) is bounded, $\varepsilon \le c(x) \le 1$ for all $x \in {\mathbb {R}}^+$. So that:

$$\begin{aligned}&\int _{0}^{\infty }\int _{y}^{y+1}P_{\infty }(y) \left( u(x) - u(y) \right) ^2 \mathrm {d} x \mathrm {d} y\\&\le \, \frac{b}{\varepsilon }e ^{\frac{1}{b}}\int _{0}^{\infty }\int _{y}^{y+1}\omega (x-y)c(y)P_{\infty }(y)\left( u(x) - u(y) \right) ^2 \mathrm {d} x \mathrm {d} y \\&\le \, \frac{b}{\varepsilon }e ^{\frac{1}{b}}\int _{0}^{\infty }\int _{y}^{\infty }\omega (x-y)c(y)P_{\infty }(y)\left( u(x) - u(y) \right) ^2 \mathrm {d} x \mathrm {d} y\\&=\, \frac{b}{a\varepsilon }e ^{\frac{1}{b}} {\mathcal {D}}_2(u), \end{aligned}$$

which proves the inequality (3.15). $\square$

#### Proof of Theorem 1.1

Putting together (3.9) and (3.15) from Propositions 3.4 and 3.5, we deduce that the entropy-entropy production inequality (3.4) holds. Lemma 3.1 together with (3.4) finally implies (3.2). As consequence, we deduce the exponential convergence towards $P_{\infty }$ for all mild solutions of (1.3). $\square$

### Numerical illustration of exponential convergence

**Fig. 4:**
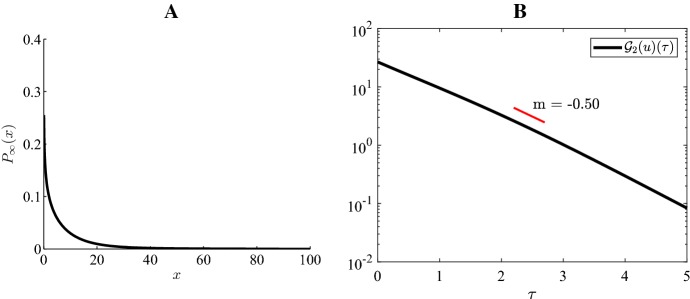
Case 1 Fig 3: $$H=-4, \ \varepsilon =0.15, \ K=45, \ a=5, \ b=10$$

**Fig. 5:**
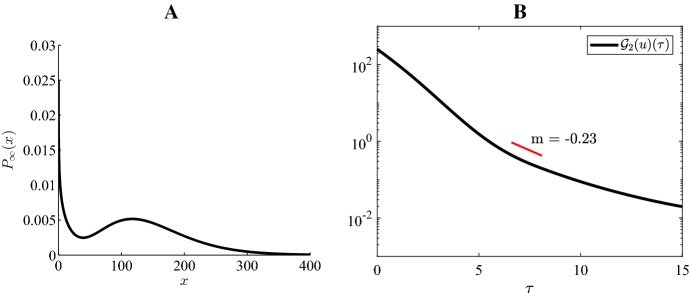
Case 2 Fig 3: $$H=-4, \ \varepsilon =0.15, \ K=45, \ a=5, \ b=30$$

**Fig. 6:**
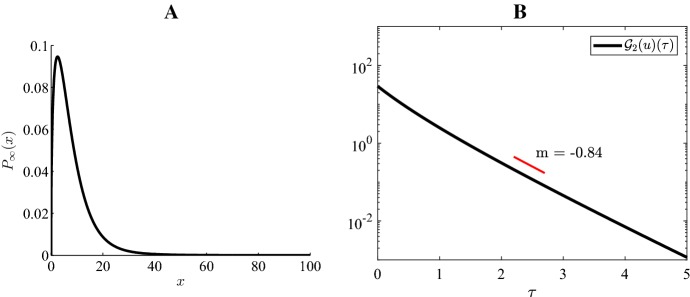
Case 3 Fig 3: $$H=-4, \ \varepsilon =0.15, \ K=45, \ a=10, \ b=5$$

**Fig. 7:**
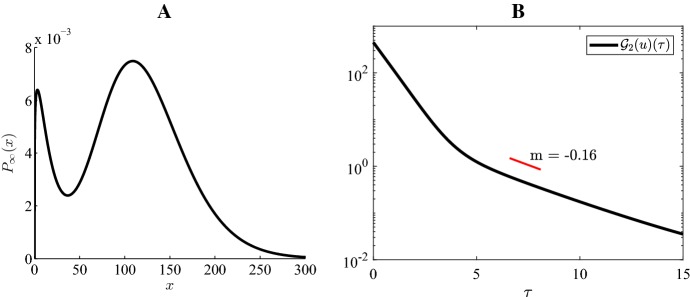
Case 4 Fig 3: $$H=-4, \ \varepsilon =0.15, \ K=45, \ a=8, \ b=16$$

**Fig. 8:**
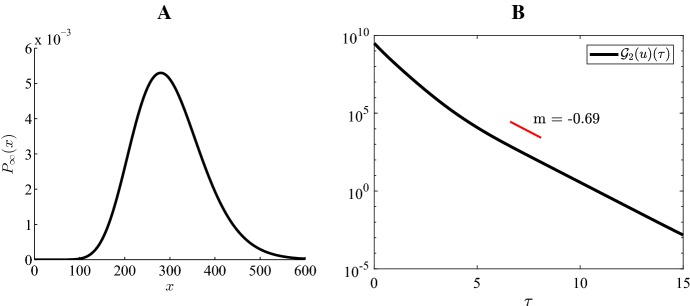
Case 5 Fig 3: $$H=-4, \ \varepsilon =0.15, \ K=45, \ a=15, \ b=20$$

The entropy functional, ${\mathcal {G}}_2(u)(t)$, is represented in the plots B of Figs. 4, 5, 6, 7 and 8, which address the five possible steady states plots A of Figs. 4, 5, 6, 7 and 8 (see also Fig. 3). For all cases, these functions are represented in a semi-logarithm scale to numerically validate the exponential convergence shown in the previous section. A Gaussian distribution with mean 2 and standard deviation 0.1, ${\mathcal {N}}(2,0.1)$, has been considered as initial condition.

## The *nD* PIDE model

We can generalise the entropy functional (2.3) defined for the one dimension PIDE model in order to study the convergence of the multidimensional model. A well-posedness theory of mild and classical solutions satisfying the positivity and mass preservation, the $L^1$-contraction principle, and the maximum principle can be analogously obtained from the one dimensional strategy in Sect. 2. Let us summarize these properties in the next proposition.

### Proposition 4.1

Given any mild solution of Eq. (1.8) with normalised initial data, then the solution satisfies

(i) Mass conservation:
  $$\begin{aligned} \int _{{{\mathbb {R}}}_{+}^{n}}p(t,{\mathbf {x}})\mathrm {d} {\mathbf {x}}=\int _{{{\mathbb {R}}}_{+}^{n}}p_0({\mathbf {x}})\mathrm {d} {\mathbf {x}} =1 \end{aligned}$$
(ii) If $p_0$ is nonnegative, then the solution *p*(*t*) of Eq. (1.8) is nonnegative for all $t\ge 0$.
(iii) $L^1$-contraction principle:
  $$\begin{aligned} \int _{{{\mathbb {R}}}_{+}^{n}}|p(t,{\mathbf {x}})|\mathrm {d} {\mathbf {x}} \le \int _{{{\mathbb {R}}}_{+}^{n}}|p_0({\mathbf {x}})|\mathrm {d} {\mathbf {x}}. \end{aligned}$$
(iv) $$L^q$$ bounds, $$1<q<\infty $$:
  $$\begin{aligned}&\int _{{{\mathbb {R}}}_{+}^{n}}P_{\infty }({\mathbf {x}})|u(t,{\mathbf {x}})|^q\mathrm {d} {\mathbf {x}}\\&\qquad \le \int _{{{\mathbb {R}}}_{+}^{n}}P_{\infty }({\mathbf {x}})|u_0({\mathbf {x}})|^q\mathrm {d} {\mathbf {x}} ~~\text {with}~~ u(t,{\mathbf {x}}):=\frac{p(t,{\mathbf {x}})}{P_{\infty }({\mathbf {x}})}\quad \text{ and }\quad u_0({\mathbf {x}}):=\frac{p_0({\mathbf {x}})}{P_{\infty }({\mathbf {x}})}. \end{aligned}$$
(v) Maximum principle:
  $$\begin{aligned} \mathop {{{\mathrm{ess\,inf}}}}\limits _{{\mathbf {x}} \in {{\mathbb {R}}}_{+}^{n}} u_0({\mathbf {x}})\le u(t,{\mathbf {x}}) \le \mathop {{{\mathrm{ess\,sup}}}}\limits _{{\mathbf {x}} \in {{\mathbb {R}}}_{+}^{n}} u_0({\mathbf {x}}). \end{aligned}$$

We will not do any details of these classical results. We just point out that these properties can be formally seen as consequences of the general relative entropy method (Michel et al. 2004, 2005). Let us now concentrate on the entropy method. Given *H*(*u*) any convex function of *u*, we define the *n*-dimensional general relative entropy functional as:

$$\begin{aligned} {\mathcal {G}}_H^n(u):= \int _{{{\mathbb {R}}}_{+}^{n}} H(u({\mathbf {x}})) P_{\infty }({\mathbf {x}}) \mathrm {d}{\mathbf {x}}, \end{aligned}$$

with $u({\mathbf {x}}):=p({\mathbf {x}})/P_\infty ({\mathbf {x}})$ as above. The main difference in the multidimensional case is that the stationary states are not explicit and thus, we need to assume certain properties on their behavior. In fact, in order to apply the entropy-entropy production method we make the following assumption:

### Assumption 4.1

The following property holds

$$\begin{aligned} \int _0^{\infty } \dfrac{\partial [H(u({\mathbf {x}}))\gamma _x^i({\mathbf {x}}) x_i P_\infty ({\mathbf {x}})]}{\partial x_i} \mathrm {d}x_i = 0 , \qquad \forall i=1,\ldots ,n, \end{aligned}$$

for any convex function *H*(*u*) and for all differentiable $p \in L^1((0,+\infty )) \cap L^2((0,+\infty ), P_\infty ^{-1})$.

Similarly to the one dimensional case, we can obtain the following identity. The proof is totally analogous to the one of Lemma 2.6 and we skip it here for brevity.

### Lemma 4.2

Let *p* be a differentiable function on ${\mathbb {R}}^n_+$. For any $i=1,\ldots ,n$ the following equality is verified:

$$\begin{aligned} H'(u({\mathbf {x}}))\dfrac{\partial [\gamma _x^i({\mathbf {x}}) x_i p({\mathbf {x}})]}{\partial x_i}&= \dfrac{\partial [H(u({\mathbf {x}}))\gamma _x^i({\mathbf {x}}) x_i P_{\infty }({\mathbf {x}})]}{\partial x_i} \\&\ \quad + \left( u({\mathbf {x}})H'(u({\mathbf {x}}))-H(u({\mathbf {x}}))\right) \dfrac{\partial [\gamma _x^i({\mathbf {x}}) x_i P_{\infty }({\mathbf {x}})]}{\partial x_i}. \end{aligned}$$

With this identity, we can now derive the evolution of the relative entropy as in the one dimensional case. We will not make explicit the time dependency of the solutions again for simplicity.

### Proposition 4.3

Let *p* be a classical solution to the *nD* PIDE model with initial data $p_0 \in L^1({\mathbb {R}}^n_+) \cap {\mathcal {C}}^1({\mathbb {R}}^n_+)$. For any convex function $H(u({\mathbf {x}}))$, the general entropy functional ${\mathcal {G}}_H^n(u)$ satisfies

4.1 $$\begin{aligned} \dfrac{\mathrm {d}{\mathcal {G}}_H^n(u)}{\mathrm {d} t}= & {} \sum _{i=1}^{n}k_m^i\! \int _{{{\mathbb {R}}}_{+}^{n}}\!\!\int _{y_i}^{\infty }\left[ H(u({\mathbf {x}})) - H(u({\mathbf {y}}_i)) +H'\left( u({\mathbf {x}}))(u({\mathbf {y}}_i)-u({\mathbf {x}})\right) \right] \nonumber \\&\quad \omega _{c,i} P_{\infty }({\mathbf {y}}_i) \mathrm {d} x_i \mathrm {d} {\mathbf {y}}_i\nonumber \\\le & {} 0, \end{aligned}$$

with the shortcut $\omega _{c,i}=\omega _i(x_i-y_i) c_i({\mathbf {y}}_i)$.

### Proof of proposition 4.3

We compute the time derivative of the general relative entropy functional to get

$$\begin{aligned} \dfrac{\mathrm {d}{\mathcal {G}}_H^n(u)}{\mathrm {d}t}&= \dfrac{\partial }{\partial t} \int _{{{\mathbb {R}}}_{+}^{n}}H(u({\mathbf {x}}))P_{\infty }({\mathbf {x}})\mathrm {d}{\mathbf {x}}\\&= \int _{{{\mathbb {R}}}_{+}^{n}}\dfrac{\partial }{\partial t} H(u({\mathbf {x}}))P_{\infty }({\mathbf {x}})\mathrm {d}{\mathbf {x}} = \int _{{{\mathbb {R}}}_{+}^{n}}H'(u({\mathbf {x}}))\dfrac{\partial p }{\partial t} \mathrm {d}{\mathbf {x}} . \end{aligned}$$

Replacing the time derivative of $p({\mathbf {x}})$ in the last equality by its expression (1.8), we obtain

$$\begin{aligned} \dfrac{\mathrm {d}{\mathcal {G}}_H^n(u)}{\mathrm {d} t}&= \int _{{{\mathbb {R}}}_{+}^{n}}H'(u({\mathbf {x}}))\left( \sum _{i=1}^{n}\left( \dfrac{\partial }{\partial x_i}\left[ \gamma _x^i({\mathbf {x}}) x_i p({\mathbf {x}})\right] \right) \right) \mathrm {d}{\mathbf {x}}\\&\quad +\int _{{{\mathbb {R}}}_{+}^{n}}H'(u({\mathbf {x}}))\left( \sum _{i=1}^{n}\left( k_m^i \int _0^{x_i} \! \omega _i(x_i-y_i) c_i({\mathbf {y}}_i)p({\mathbf {y}}_i) \, \mathrm {d}y_i -k_1^ic_i({\mathbf {x}})p({\mathbf {x}})\right) \right) \mathrm {d}{\mathbf {x}}. \end{aligned}$$

Summations and integrals in the above expression are interchangeable, so that

4.2 $$\begin{aligned} \dfrac{\mathrm {d}{\mathcal {G}}_H^n(u)}{\mathrm {d} t}=&\,\sum _{i=1}^{n}\left( \int _{{{\mathbb {R}}}_{+}^{n}}H'(u({\mathbf {x}}))\left( \dfrac{\partial }{\partial x_i}\left[ \gamma _x^i({\mathbf {x}}) x_i p({\mathbf {x}})\right] \right) \right) \mathrm {d}{\mathbf {x}} \nonumber \\&+\sum _{i=1}^{n}\left( \int _{{{\mathbb {R}}}_{+}^{n}}H'(u({\mathbf {x}}))\left( k_m^i \int _0^{x_i} \! \omega _i(x_i-y_i) c_i({\mathbf {y}}_i)p({\mathbf {y}}_i) \, \mathrm {d}y_i -k_1^ic_i({\mathbf {x}})p({\mathbf {x}})\right) \right) \mathrm {d}{\mathbf {x}}. \end{aligned}$$

Next, using Lemma 4.2, the first term on the right hand side in the above equation becomes

4.3 $$\begin{aligned}&\sum _{i=1}^{n} \left( \int _{{{\mathbb {R}}}_{+}^{n}} \right. \left. H'(u({\mathbf {x}}))\left( \dfrac{\partial }{\partial x_i}\left[ \gamma _x^i({\mathbf {x}}) x_i p({\mathbf {x}})\right] \right) \right) \mathrm {d}{\mathbf {x}} \nonumber \\&\quad =\sum _{i=1}^{n}\left( \int _{{{\mathbb {R}}}_{+}^{n}} \dfrac{\partial [H(u({\mathbf {x}}))\gamma _x^i({\mathbf {x}}) x_i P_{\infty }({\mathbf {x}})]}{\partial x_i}\nonumber \right. \\&\qquad \left. + \left( u({\mathbf {x}})H'(u({\mathbf {x}}))-H(u({\mathbf {x}}))\right) \dfrac{\partial [\gamma _x^i({\mathbf {x}}) x_i P_{\infty }({\mathbf {x}})]}{\partial x_i} \right) \mathrm {d}{\mathbf {x}} \nonumber \\&\quad =\sum _{i=1}^{n}\left( \int _{{{\mathbb {R}}}_{+}^{n}} \left( u({\mathbf {x}})H'(u({\mathbf {x}}))-H(u({\mathbf {x}}))\right) \dfrac{\partial [\gamma _x^i({\mathbf {x}}) x_i P_{\infty }({\mathbf {x}})]}{\partial x_i} \right) \mathrm {d}{\mathbf {x}} , \end{aligned}$$

this last identity holds using Assumption 4.1. Note that, the first term in the last summation in Eq. (4.3) is equivalent to

4.4 $$\begin{aligned} \sum _{i=1}^{n}&\left( \int _{{{\mathbb {R}}}_{+}^{n}} u({\mathbf {x}})H'(u({\mathbf {x}}))\dfrac{\partial [\gamma _x^i({\mathbf {x}}) x_i P_{\infty }({\mathbf {x}})]}{\partial x_i} \right) \mathrm {d}{\mathbf {x}} \nonumber \\&= \int _{{{\mathbb {R}}}_{+}^{n}} u({\mathbf {x}})H'(u({\mathbf {x}}))\sum _{i=1}^{n}\left( \dfrac{\partial [\gamma _x^i({\mathbf {x}}) x_i P_{\infty }({\mathbf {x}})]}{\partial x_i} \right) \mathrm {d}{\mathbf {x}} \nonumber \\&=\int _{{{\mathbb {R}}}_{+}^{n}}u({\mathbf {x}})H'(u({\mathbf {x}}))\left( \sum _{i=1}^{n}\left( - k_m^i \int _0^{x_i} \! \omega _i(x_i-y_i) c_i({\mathbf {y}}_i)P_{\infty }({\mathbf {y}}_i) \, \mathrm {d}y_i + k_1^ic_i({\mathbf {x}})P_{\infty }({\mathbf {x}})\right) \right) \mathrm {d}{\mathbf {x}} \nonumber \\&=\sum _{i=1}^{n}\left( \int _{{{\mathbb {R}}}_{+}^{n}}H'(u({\mathbf {x}}))\left( - u({\mathbf {x}})k_m^i \int _0^{x_i} \! \omega _i(x_i-y_i) c_i({\mathbf {y}}_i)P_{\infty }({\mathbf {y}}_i) \, \mathrm {d}y_i + k_1^ic_i({\mathbf {x}})p({\mathbf {x}})\right) \right) \mathrm {d}{\mathbf {x}}, \end{aligned}$$

and the second term in the last summation in Eq. (4.3) is equivalent to

4.5 $$\begin{aligned} \sum _{i=1}^{n}&\left( \int _{{{\mathbb {R}}}_{+}^{n}} -H(u({\mathbf {x}}))\dfrac{\partial [\gamma _x^i({\mathbf {x}}) x_i P_{\infty }({\mathbf {x}})]}{\partial x_i} \right) \mathrm {d}{\mathbf {x}} = \int _{{{\mathbb {R}}}_{+}^{n}}- H(u({\mathbf {x}}))\sum _{i=1}^{n}\left( \dfrac{\partial [\gamma _x^i({\mathbf {x}}) x_i P_{\infty }({\mathbf {x}})]}{\partial x_i} \right) \mathrm {d}{\mathbf {x}}\nonumber \\&=\int _{{{\mathbb {R}}}_{+}^{n}}-H(u({\mathbf {x}}))\left( \sum _{i=1}^{n}\left( - k_m^i \int _0^{x_i} \! \omega _i(x_i-y_i) c_i({\mathbf {y}}_i)P_{\infty }({\mathbf {y}}_i) \, \mathrm {d}y_i + k_1^ic_i({\mathbf {x}})P_{\infty }({\mathbf {x}})\right) \right) \mathrm {d}{\mathbf {x}} \nonumber \\&=\sum _{i=1}^{n}\left( \int _{{{\mathbb {R}}}_{+}^{n}}H(u({\mathbf {x}}))\left( k_m^i \int _0^{x_i} \! \omega _i(x_i-y_i) c_i({\mathbf {y}}_i)P_{\infty }({\mathbf {y}}_i) \, \mathrm {d}y_i - k_1^ic_i({\mathbf {x}})P_{\infty }({\mathbf {x}})\right) \right) \mathrm {d}{\mathbf {x}}. \end{aligned}$$

Thus, using the expressions (4.4–4.5), replacing first in (4.3) and finally in the Eq. (4.2), we obtain the following equality

4.6 $$\begin{aligned} \dfrac{\mathrm {d}{\mathcal {G}}_H^n(u)}{\mathrm {d} t}&= \sum _{i=1}^{n}\left( \int _{{{\mathbb {R}}}_{+}^{n}}\left( - u(t,{\mathbf {x}})k_m^i \int _0^{x_i} \! \omega _i(x_i-y_i) c_i({\mathbf {y}}_i)P_{\infty }({\mathbf {y}}_i) \, \mathrm {d}y_i + k_1^ic_i({\mathbf {x}})p({\mathbf {x}})\right) H'(u({\mathbf {x}}))\right) \mathrm {d}{\mathbf {x}} \nonumber \\&\quad + \sum _{i=1}^{n}\left( \int _{{{\mathbb {R}}}_{+}^{n}}H(u({\mathbf {x}}))\left( k_m^i \int _0^{x_i} \! \omega _i(x_i-y_i) c_i({\mathbf {y}}_i)P_{\infty }({\mathbf {y}}_i) \, \mathrm {d}y_i - k_1^ic_i({\mathbf {x}})P_{\infty }({\mathbf {x}})\right) \right) \mathrm {d}{\mathbf {x}} \nonumber \\&\quad +\sum _{i=1}^{n}\left( \int _{{{\mathbb {R}}}_{+}^{n}}H'(u({\mathbf {x}}))\left( k_m^i \int _0^{x_i} \! \omega _i(x_i-y_i) c_i({\mathbf {y}}_i)p(t,{\mathbf {y}}_i) \, \mathrm {d}y_i -k_1^ic_i({\mathbf {x}})p({\mathbf {x}})\right) \right) \mathrm {d}{\mathbf {x}}\nonumber \\&=\, \sum _{i=1}^{n}\left( \int _{{{\mathbb {R}}}_{+}^{n}}H(u({\mathbf {x}}))\left( k_m^i \int _0^{x_i} \! \omega _i(x_i-y_i) c_i({\mathbf {y}}_i)P_{\infty }({\mathbf {y}}_i) \, \mathrm {d}y_i - k_1^ic_i({\mathbf {x}})P_{\infty }({\mathbf {x}})\right) \right) \mathrm {d}{\mathbf {x}} \nonumber \\&\quad +\sum _{i=1}^{n}\left( \int _{{{\mathbb {R}}}_{+}^{n}}\left( k_m^i \int _0^{x_i} \! \omega _i(x_i-y_i) c_i({\mathbf {y}}_i)P_{\infty }({\mathbf {y}}_i)\left[ u(t,{\mathbf {y}}_i)-u(t,{\mathbf {x}})\right] \, \mathrm {d}y_i \right) H'(u({\mathbf {x}}))\right) \mathrm {d}{\mathbf {x}}. \end{aligned}$$

By changing the order of integration in the above expression and using the following identity

$$\begin{aligned} \int _{y_i}^{\infty }\omega _i(x_i-y_i)\mathrm {d}x_i=1, \qquad \forall i=1,\ldots ,n, \end{aligned}$$

the Eq. (4.6) can be rewritten in the following equivalent form

$$\begin{aligned}&\dfrac{\mathrm {d}{\mathcal {G}}_H^n(u)}{\mathrm {d} t}= \, \sum _{i=1}^{n}\left( k_m^i\int _{{{\mathbb {R}}}_{+}^{n}} \int _{y_i}^{\infty } \! \omega _i(x_i-y_i) c_i({\mathbf {y}}_i)P_{\infty }({\mathbf {y}}_i) \left[ H(u({\mathbf {x}})) -H(u(t,{\mathbf {y}}_i))\right] \, \mathrm {d}x_i \right) \mathrm {d}{\mathbf {y}}_i\\&\quad \,+\sum _{i=1}^{n}\left( \int _{{{\mathbb {R}}}_{+}^{n}}\left( k_m^i \int _{y_i}^{\infty } \! \omega _i(x_i-y_i) c_i({\mathbf {y}}_i)P_{\infty }({\mathbf {y}}_i)H'(u({\mathbf {x}}))\left[ u(t,{\mathbf {y}}_i)-u(t,{\mathbf {x}})\right] \, \mathrm {d}x_i \right) \right) \mathrm {d}{\mathbf {y}}_i, \end{aligned}$$

which is equivalent to the expression (4.1) defined in Proposition 4.3, thus concluding the derivation of the identity. Observe finally that due to the convexity of *H*(*u*), we deduce that $H(u)-H(v)+H'(u)(v-u) \le 0$ for all *u*, *v* leading to final claim. $\square$

As in the one dimensional case, we will focus on the $L^2$-relative entropy, i.e., we choose $H(u)=(u-1)^2$ to define

$$\begin{aligned} {\mathcal {G}}_2^n(u):=\int _{{{\mathbb {R}}}_{+}^{n}}\left( u({\mathbf {x}}) -1\right) ^2 P_{\infty } \mathrm {d}{\mathbf {x}} \end{aligned}$$

and

$$\begin{aligned} {\mathcal {D}}_2^n(u)= \sum _{i=1}^{n}k_m^i\int _{{{\mathbb {R}}}_{+}^{n}}\int _{y_i}^{\infty }\omega _i(x_i-y_i)\left[ u({\mathbf {x}}) - u({\mathbf {y}}_i) \right] ^2 c_i({\mathbf {y}}_i)P_\infty ({\mathbf {y}}_i) \mathrm {d} x_i \mathrm {d} {\mathbf {y}}_i . \end{aligned}$$

Proposition 4.3 leads to the relation

4.7 $$\begin{aligned} \dfrac{\mathrm {d}{\mathcal {G}}_2^n(u)}{\mathrm {d} t} =-{\mathcal {D}}_2^n(u)\le 0. \end{aligned}$$

### Approach to equilibrium

Based on the Assumption 4.1 on stationary solutions, we are now able to control the entropy by the entropy production except for a small error term.

#### Lemma 4.4

Assume that $p \le C_1 P_\infty$ for some $C_1 > 0$. Then, for each $\epsilon > 0$ there exists a constant $K_\epsilon > 0$ depending on $C_1$ and $\epsilon$ such that:

$$\begin{aligned} {\mathcal {G}}_2^n(u) \le K_\epsilon {\mathcal {D}}_2^n(u) + \epsilon . \end{aligned}$$

#### Proof

By expanding the square, we can write

4.8 $$\begin{aligned} {\mathcal {G}}_2^n(u) = \frac{1}{2} \int _{{\mathbb {R}}^n_+} \int _{{\mathbb {R}}^n_+} P_\infty ({\mathbf {x}}) P_\infty ({\mathbf {y}}) (u(t,{\mathbf {x}}) - u({\mathbf {y}}))^2 \,\mathrm {d}{\mathbf {x}} \,\mathrm {d}{\mathbf {y}}. \end{aligned}$$

We split the latter integral in two parts: the integral over $\Omega _\delta \times \Omega _\delta$, and the integral over its complement with

$$\begin{aligned} \Omega _{\delta }=\overbrace{\left[ \delta , \ 1/\delta \right] \times \dots \times \left[ \delta , \ 1/\delta \right] }^{n \text { times}} \text { such that, } \delta \in (0, \ 1). \end{aligned}$$

For the integral over the complement, using $p \le C_1 P_\infty$, we deduce

$$\begin{aligned}&\iint _{{\mathbb {R}}^{2n}_+ \setminus (\Omega _\delta \times \Omega _\delta )} P_\infty ({\mathbf {x}}) P_\infty ({\mathbf {y}}) (u({\mathbf {x}}) - u({\mathbf {y}}))^2 \,\mathrm {d}{\mathbf {x}} \,\mathrm {d}{\mathbf {y}}\\&\quad \le 2 C_1^2 \iint _{{\mathbb {R}}^{2n}_+ \setminus (\Omega _\delta \times \Omega _\delta )} P_\infty ({\mathbf {x}}) P_\infty ({\mathbf {y}}) \,\mathrm {d}{\mathbf {x}} \,\mathrm {d}{\mathbf {y}}. \end{aligned}$$

On the other hand, for the integral over $\Omega _\delta \times \Omega _\delta$ we get

$$\begin{aligned} \int _{\Omega _\delta } \int _{\Omega _\delta } P_\infty ({\mathbf {x}}) P_\infty ({\mathbf {y}}) (u({\mathbf {x}}) - u({\mathbf {y}}))^2 \,\mathrm {d}{\mathbf {x}} \,\mathrm {d}{\mathbf {y}} \le K_{\delta ,1} \int _{\Omega _\delta } \int _{\Omega _\delta } (u({\mathbf {x}}) - u({\mathbf {y}}))^2 \,\mathrm {d}{\mathbf {x}} \,\mathrm {d}{\mathbf {y}}, \end{aligned}$$

where

$$\begin{aligned} K_{\delta ,1} := \sup _{({\mathbf {x}}, {\mathbf {y}}) \in \Omega _\delta \times \Omega _\delta } P_\infty ({\mathbf {x}}) P_\infty ({\mathbf {y}}) < +\infty . \end{aligned}$$

We now rewrite $u({\mathbf {x}}) - u({\mathbf {y}})$ as a sum of *n* terms, each of which being a difference of values of *u* at points which differ only by one coordinate

$$\begin{aligned} u({\mathbf {x}}) - u({\mathbf {y}}) = \sum _{i=1}^n \Big ( u(x_1, \dots , x_i, y_{i+1}, \dots , y_n) - u(x_1, \dots , x_{i-1}, y_{i}, \dots , y_n) \Big ), \end{aligned}$$

(where it is understood that $$u(x_1, \dots , x_i, y_{i+1}, \dots , y_n) = u({\mathbf {x}})$$ for $$i=n$$, and $$u(x_1, \dots ,$$$$ x_{i-1}, y_{i}, \dots , y_n) = u({\mathbf {y}})$$ for $$i=1$$). Then, by Cauchy-Schwarz’s inequality we have

$$\begin{aligned}&\int _{\Omega _\delta } \int _{\Omega _\delta } (u({\mathbf {x}}) - u({\mathbf {y}}))^2 \,\mathrm {d}{\mathbf {x}} \,\mathrm {d}{\mathbf {y}} \\&\quad \le n \sum _{i=1}^n \int _{\Omega _\delta } \int _{\Omega _\delta } \Big ( u(x_1, \dots , x_i, y_{i+1}, \dots , y_n) - u(x_1, \dots , x_{i-1}, y_{i}, \dots , y_n) \Big )^2 \,\mathrm {d}{\mathbf {x}} \,\mathrm {d}{\mathbf {y}} \\&\quad = n \left( \frac{1}{\delta } - \delta \right) ^{n-1} \sum _{i=1}^n \int _{[\delta , 1/\delta ]^n} \int _{[\delta , 1/\delta ]} \Big ( u({\mathbf {x}}) - u({\mathbf {y}}_i) \Big )^2 \,\mathrm {d}x_i \,\mathrm {d}{\mathbf {y}}_i \\&\quad = 2n \left( \frac{1}{\delta } - \delta \right) ^{n-1} \sum _{i=1}^n \int _{[\delta , 1/\delta ]^n} \int _{y_i}^{1/\delta } \Big ( u({\mathbf {x}}) - u({\mathbf {y}}_i) \Big )^2 \,\mathrm {d}x_i \,\mathrm {d}{\mathbf {y}}_i \\&\quad \le K_{\delta ,2} \sum _{i=1}^n k_m^i \int _{[\delta , 1/\delta ]^n} \int _{y_i}^{1/\delta } \omega _i(x_i-y_i) c_i({\mathbf {y}}_i) P_\infty ({\mathbf {y}}_i) \Big ( u({\mathbf {x}}) - u({\mathbf {y}}_i) \Big )^2 \,\mathrm {d}x_i \,\mathrm {d}{\mathbf {y}}_i, \end{aligned}$$

therefore we conclude that

4.9 $$\begin{aligned} \int _{\Omega _\delta } \int _{\Omega _\delta } (u({\mathbf {x}}) - u({\mathbf {y}}))^2 \,\mathrm {d}{\mathbf {x}} \,\mathrm {d}{\mathbf {y}} \le K_{\delta ,2} {\mathcal {D}}_2(p), \end{aligned}$$

where $K_{\delta ,2}$ is defined by

$$\begin{aligned} 2 n \left( \frac{1}{\delta } - \delta \right) ^{n-1} K_{\delta ,2}^{-1} = \inf \big ( k_m^i \omega _i(x_i-y_i) c_i({\mathbf {y}}_i) P_\infty ({\mathbf {y}}_i) \big ), \end{aligned}$$

with the infimum running over all $i = 1, \dots , n$ and over all the points in the domain of integration. We notice that the first of the equalities in (4.9) is just obtained by integrating in the variables that do not appear in the expression and renaming the others; and the second equality is due to the symmetry of the integrand in the variables $(x_i, y_i)$. Using (4.8)–(4.9) finally gives:

$$\begin{aligned} {\mathcal {G}}_2^n(u) \le C_1^2 \iint _{{\mathbb {R}}^{2n}_+ \setminus (\Omega _\delta \times \Omega _\delta )} P_\infty ({\mathbf {x}}) P_\infty ({\mathbf {y}}) \,\mathrm {d}{\mathbf {x}} \,\mathrm {d}{\mathbf {y}} + \frac{1}{2} K_{\delta ,1} K_{\delta ,2} {\mathcal {D}}_2^n(u). \end{aligned}$$

We may choose $\delta > 0$ such that the first term is smaller than $\epsilon$. This gives then the result with $K_\epsilon = \frac{1}{2} K_{\delta ,1} K_{\delta ,2}$. $\square$

#### Theorem 4.5

(Long-time behaviour) Given any mild solution *p* with normalised nonnegative initial data $p_0 \in L^1({\mathbb {R}}_+)$ to Eq. (1.8) and given a stationary solution $P_\infty ({\mathbf {x}})$ to (1.8) satisfying Assumption 4.1, then

$$\begin{aligned} \lim _{t \rightarrow \infty } \int _{{{\mathbb {R}}}_{+}^{n}}|p(t,{\mathbf {x}})-P_\infty ({\mathbf {x}})|^2 \mathrm {d} {\mathbf {x}} = 0. \end{aligned}$$

As a consequence, stationary solutions $P_\infty ({\mathbf {x}})$ of (1.8) satisfying Assumption  4.1, if they exist, they are unique.

#### Proof

**Step 1: Proof for “nice” initial data.** We first prove the result for initial data $p_0 \in L^1({\mathbb {R}}^n_+) \cap {\mathcal {C}}^2({\mathbb {R}}^n_+)$ such that $p_0 \le C_1 P_\infty$, for some constant $C_1 > 0$. Observe that this implies in particular that $p_0 \in L^2({\mathbb {R}}^n_+, P_\infty ({\mathbf {x}})^{-1} \,\mathrm {d}{\mathbf {x}})$. For such initial data we deduce that for all $t \ge 0$

$$\begin{aligned} p(t,{\mathbf {x}}) \le C_1 P_\infty ({\mathbf {x}}) \quad \text { for almost all}~ {\mathbf {x}} \in {\mathbb {R}}^n_+, \end{aligned}$$

from the maximum principle. This enables us to use Lemma 4.4. Using the general entropy identity with $H(u)=(u-1)^2$, from Proposition 4.3 we obtain:

4.10 $$\begin{aligned} \dfrac{\mathrm {d} {\mathcal {G}}_2^n(u)}{\mathrm {d} t} = -{\mathcal {D}}_2^n(u). \end{aligned}$$

Next, by using time integration on [0, *T*] in Eq. (4.10), the following equality holds for all $T>0$:

$$\begin{aligned} {\mathcal {G}}_2^n(u)(T) + \int _0^T {\mathcal {D}}_2^n(p)(t) \,\mathrm {d}t = {\mathcal {G}}_2^n(u)(0), \end{aligned}$$

from which we deduce that:

4.11 $$\begin{aligned} \int _0^\infty {\mathcal {D}}_2^n(u)(t) \,\mathrm {d}t < \infty . \end{aligned}$$

From (4.11), there exists a sequence $(t_s)_{s \ge 1}$ such that ${\mathcal {D}}_2^n(u)(t_s) \rightarrow 0$ as $s \rightarrow +\infty$. Thus if we take any $\epsilon > 0$, then Lemma 4.4 gives:

$$\begin{aligned} {\mathcal {G}}_2^n(u)(t_s) \le K_\epsilon {\mathcal {D}}_2^n(u)(t_s) + \epsilon \rightarrow \epsilon \quad \text {as}~ {s \rightarrow +\infty }. \end{aligned}$$

Since ${\mathcal {G}}_2^n(u)(t)$ is decreasing in *t*, this shows that $\lim _{t \rightarrow +\infty } {\mathcal {G}}_2^n(u)(t) \le \epsilon$. Since $\epsilon$ is arbitrary chosen, we deduce that:

$$\begin{aligned} {\mathcal {G}}_2^n(u)(t) \rightarrow 0 \quad \text {as}~ {t \rightarrow +\infty }. \end{aligned}$$

**Step 2: Proof for all integrable initial data.** It is now classical to extend the result in step 1 to all initial data in $L^1({\mathbb {R}}^n_+)$ by the $L^1$-contraction principle. In fact, any $p_0 \in L^1({\mathbb {R}}^n_+)$ can be approximated in $L^1({\mathbb {R}}^n_+)$ by a sequence $(p_0^s)_{s \ge 1}$ such that $p_0^s \le s P_\infty$, for all $s \ge 1$. Thus consider the solution $p^s$ associated to initial data $p_0^s$. By step 1, we get

$$\begin{aligned} \int _0^\infty |p^s(t, {\mathbf {x}}) - P_\infty ({\mathbf {x}})| \,\mathrm {d}{\mathbf {x}} \rightarrow 0 \quad \text {as}~ {t \rightarrow +\infty }, \end{aligned}$$

since ${\mathcal {G}}_2^n(u_s)(t) \ge \Vert p^s(t, {\mathbf {x}}) - P_\infty ({\mathbf {x}})\Vert _1^2$ with $u^s=\frac{p^s}{P_\infty }$. Hence, for $s \ge 1$ we deduce

$$\begin{aligned} \int _0^\infty |p(t, {\mathbf {x}}) - P_\infty ({\mathbf {x}})| \,\mathrm {d}{\mathbf {x}}&\le \int _0^\infty |p(t, {\mathbf {x}}) - p^s(t, {\mathbf {x}})| \,\mathrm {d}{\mathbf {x}} + \int _0^\infty | p^s(t, {\mathbf {x}}) - P_\infty ({\mathbf {x}})| \,\mathrm {d}{\mathbf {x}} \nonumber \\&\le \int _0^\infty |p_0({\mathbf {x}}) - p_0^s({\mathbf {x}})| \,\mathrm {d}{\mathbf {x}} + \int _0^\infty | p^s(t, {\mathbf {x}}) - P_\infty ({\mathbf {x}})| \,\mathrm {d}{\mathbf {x}}, \end{aligned}$$

from the $L^1$-contraction principle. This easily leads to the result since

$$\begin{aligned} \lim _{s \rightarrow \infty } \int \nolimits _0^\infty |p_0({\mathbf {x}}) - p_0^s({\mathbf {x}})| \,\mathrm {d}{\mathbf {x}}=0 \qquad \text{ and } \qquad \lim _{t \rightarrow \infty } \int \nolimits _0^\infty | p^s(t, {\mathbf {x}}) - P_\infty ({\mathbf {x}})| \,\mathrm {d}{\mathbf {x}}=0, \end{aligned}$$

for all $$s\ge 1$$. $$\square $$

### Numerical exploration of the convergence rates

**Fig. 9:**
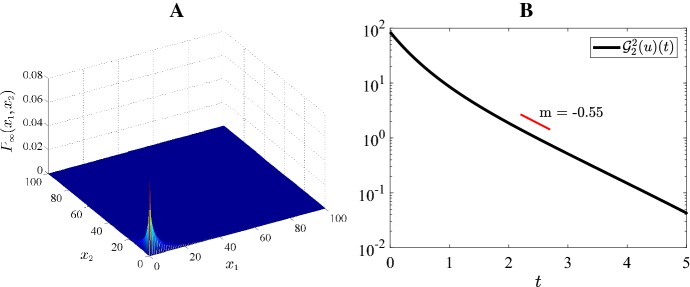
Example of two self regulated proteins whose distribution has a peak in ${\mathbf {x}}=(0,0)$. (Same parameters as in the example depicted in Fig. 4 for both proteins)

**Fig. 10:**
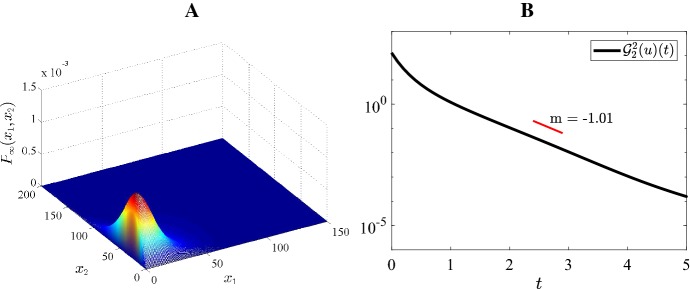
Example of two self and cross regulated proteins whose distribution has a peak in some positive point ${\mathbf {x}}=(x_1,x_2)$ with $x_1>0$ and $x_2>0$. Parameters: $\gamma _x^1=\gamma _x^2=1$, $\gamma _m^1=\gamma _m^2=25$, $k_m^1=10$, $k_m^2=20$, $b_1=10$, $b_2=20$ and input functions in (4.12)

**Fig. 11:**
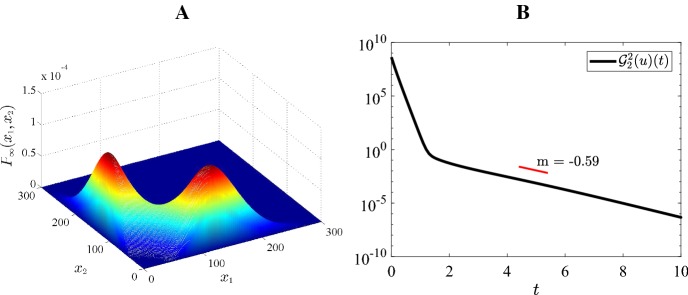
Example of two mutual repressed proteins whose joint distribution is bimodal attaining two peaks in two positive points. Parameters:$\gamma _x^1=\gamma _x^2=1$, $\gamma _m^1=\gamma _m^2=25$, $k_m^1=k_m^2=8$ and $b_1=b_2=16$ with input functions defined in (4.13)

The entropy functional, ${\mathcal {G}}_2^n(u)(t)$, is represented in the plots B of Figs. 9, 10 and 11, which address three possible steady states (plots A of Figs. 9, 10 and 11) that have been obtained using the SELANSI toolboox (Pájaro et al. 2018). For all cases, these functions are represented in a semi-logarithm scale to numerically check if the convergence shown in the previous section is exponential in higher dimensions.

In the first example, Fig. 9, we consider two different self-regulated proteins with input functions:

$$\begin{aligned} c_i(x_i)=\frac{K_i^{H_i} + \varepsilon _i x_i^{H_i}}{K_i^{H_i} + x_i^{H_i}} \quad \text {for}~ {i=1,2}, \end{aligned}$$

with $H_i=-4$, $\varepsilon _i=0.15$, $K_i=45$, $a_i=5$ and $b_i=10$ as in the example depicted in Fig. 4.

The second example, Fig. 10, is a self and cross-regulated gene network expressing two different proteins where the first one activates the production of both itself and the second protein, while the second protein inhibits the expression of both proteins. The input functions considered, as in Pájaro et al. (2017), read:

4.12 $$\begin{aligned} \begin{array}{rl} c_1({\mathbf {x}})= &{} \dfrac{\epsilon _{11}x_1^{H_{11}}x_2^{H_{12}} + \epsilon _{12}K_{11}^{H_{11}}x_2^{H_{12}} + \epsilon _{13}x_1^{H_{11}}K_{12}^{H_{12}} + K_{11}^{H_{11}}K_{12}^{H_{12}}}{x_1^{H_{11}}x_2^{H_{12}} + K_{11}^{H_{11}}x_2^{H_{12}} + x_1^{H_{11}}K_{12}^{H_{12}} + K_{11}^{H_{11}}K_{12}^{H_{12}}}, \\ &{}\\ c_2({\mathbf {x}})= &{} \dfrac{\epsilon _{21}x_2^{H_{22}}x_1^{H_{21}} + \epsilon _{22}K_{22}^{H_{22}}x_1^{H_{21}} + \epsilon _{23}x_2^{H_{22}}K_{21}^{H_{21}} + K_{22}^{H_{22}}K_{21}^{H_{21}}}{x_2^{H_{22}}x_1^{H_{21}} + K_{22}^{H_{22}}x_1^{H_{21}} + x_2^{H_{22}}K_{21}^{H_{21}} + K_{22}^{H_{22}}K_{21}^{H_{21}}}, \end{array} \end{aligned}$$

with $H_{11}=-4$, $H_{21}=-6$, $H_{12}=H_{22}=2$, $K_{11}=K_{12}=45$, $K_{21}=K_{22}=70$, $\varepsilon _{11}=\varepsilon _{21}=0.002$, $\varepsilon _{12}=0.02$, $\varepsilon _{22}=0.1$, $\varepsilon _{13}=\varepsilon _{23}=0.2$ and network parameters $\gamma _x^1=\gamma _x^2=1$, $\gamma _m^1=\gamma _m^2=25$, $k_m^1=10$, $k_m^2=20$, $b_1=10$ and $b_2=20$.

Our third example, Fig. 11, corresponds to a mutual repressing network of two genes in which the protein produced by the expression of one gene inhibits the production of the other protein in the network. The input functions, as in Pájaro et al. (2017), for this example take the following form:

4.13 $$\begin{aligned} c_1({\mathbf {x}})= \dfrac{K_1^{H_{12}}+\varepsilon _1 x_2^{H_{12}}}{K_1^{H_{12}}+x_2^{H_{12}}}, \qquad c_2({\mathbf {x}})= \dfrac{K_2^{H_{21}}+\varepsilon _2 x_1^{H_{21}}}{K_2^{H_{21}}+x_1^{H_{21}}}, \end{aligned}$$

with $H_{12}=H_{21}=4$, $K_{1}=K_{2}=45$ and $\varepsilon _{1}=\varepsilon _{2}=0.15$. The dimensionless network parameters are $\gamma _x^1=\gamma _x^2=1$, $\gamma _m^1=\gamma _m^2=25$, $k_m^1=k_m^2=8$ and $b_1=b_2=16$.

For each example described above, a multivariate Gaussian distribution with means 10 and standard deviations 1, ${\mathcal {N}}([10, \ 10], [1, \ 1])$, has been considered as initial condition.

## Conclusions

Analytical results for the *nD* model show convergence to equilibrium via a very general method, but do not give a bound on the convergence rate. The numerical simulations we have carried out clearly support the idea that exponential convergence also holds in the multidimensional case, though we have not been able to prove this using the same entropy method as in the one-dimensional case. Approach to equilibrium seems to follow a steady exponential speed, being quickly dominated by the spectral gap expected from our analysis. There also seem to be initial regimes where the approach to equilibrium can occur much faster; our interpretation is that smaller (more negative) eigenvalues can dominate at initial stages of time evolution, but are overcome by the dominant eigenvalue as equilibrium is approached.

## References

[CR1] Alon U (2007). An introduction to systems biology. Design principles of biological circuits.

[CR2] Balagué D, Cañizo JA, Gabriel P (2013). Fine asymptotics of profiles and relaxation to equilibrium for growth-fragmentation equations with variable drift rates. Kinet Relat Models.

[CR3] Bokes P, Singh A (2015). Protein copy number distributions for a self-regulating gene in the presence of decoy binding sites. PLoS ONE.

[CR4] Bokes P, Singh A (2017). Gene expression noise is affected differentially by feedback in burst frequency and burst size. J Math Biol.

[CR5] Bokes P, Lin YT, Singh A (2018). High cooperativity in negative feedback can amplify noisy gene expression. Bull Math Biol.

[CR6] Cáceres MJ, Cañizo JA, Mischler S (2011). Rate of convergence to an asymptotic profile for the self-similar fragmentation and growth-fragmentation equations. J Math Pures Appl.

[CR7] Cañizo JA, Carrillo JA, Cuadrado SL (2013). Measure solutions for some models in population dynamics. Acta Appl Math.

[CR8] Carrillo JA, Cordier S, Mancini S (2011). A decision-making fokker-planck model in computational neuroscience. J Math Biol.

[CR9] Dar RD, Razooky BS, Singh A, Trimeloni TV, McCollum JM, Cox CD, Simpson ML, Weinberger LS (2012). Transcriptional burst frequency and burst size are equally modulated across the human genome. Proc Natl Acad Sci USA.

[CR10] Doumic Jauffret M, Gabriel P (2010). Eigenelements of a general aggregation-fragmentation model. Math Models Methods Appl Sci.

[CR11] Elgart V, Jia T, Fenley AT, Kulkarni R (2011). Connecting protein and mRNA burst distributions for stochastic models of gene expression. Phys Biol.

[CR12] Elowitz MB, Levine AJ, Siggia ED, Swain PS (2002). Stochastic gene expression in a single cell. Science.

[CR13] Engblom S (2006). Computing the moments of high dimensional solutions of the master equation. Appl Math Comput.

[CR14] Engel K-J, Nagel R (2006). A short course on operator semigroups.

[CR15] Friedman N, Cai L, Xie XS (2006). Linking stochastic dynamics to population distribution: an analytical framework of gene expression. Phys Rev Lett.

[CR16] Gillespie DT (1976). A general method for numerically simulating the stochastic time evolution of coupled chemical reactions. J Comput Phys.

[CR17] Gillespie DT (2007). Stochastic simulation of chemical kinetics. Annu Rev Phys Chem.

[CR18] Gualdani MP, Mischler S, Mouhot C (2010) Factorization for non-symmetric operators and exponential h-theorem. June

[CR19] Hasenauer J, Wolf V, Kazeroonian A, Theis FJ (2015). Method of conditional moments (mcm) for the chemical master equation: a unified framework for the method of moments and hybrid stochastic-deterministic models. J Math Biol..

[CR20] Jahnke T (2011). On reduced models for the chemical master equation. Multiscale Model. Simul..

[CR21] Kærn M, Elston TC, Blake WJ, Collins JJ (2005). Stochasticity in gene expression: from theories to phenotypes. Nat Rev Genet.

[CR22] Kepler TB, Elston TC (2001). Stochasticity in transcriptional regulation: origins, consequences, and mathematical representations. Biophys J.

[CR23] Laurençot P, Perthame B (2009). Exponential decay for the growth-fragmentation/cell-division equation. Commun Math Sci.

[CR24] Lee TH, Maheshri N (2012). A regulatory role for repeated decoy transcription factor binding sites in target gene expression. Mol Syst Biol.

[CR25] Mackey MC, Tyran-Kaminska M, Yvinec R (2011). Molecular distributions in gene regulatory dynamics. J Theor Biol.

[CR26] McAdams H, Arkin A (1997). Stochastic mechanisms in gene expression. Proc Natl Acad Sci USA.

[CR27] Michel P, Mischler S, Perthame B (2004). General entropy equations for structured population models and scattering. C R Math.

[CR28] Michel P, Mischler S, Perthame B (2005). General relative entropy inequality: an illustration on growth models. J Math Pures Appl.

[CR29] Munsky B, Khammash M (2006). The finite state projection algorithm for the solution of the chemical master equation. J Chem Phys.

[CR30] Ochab-Marcinek A, Tabaka M (2015). Transcriptional leakage versus noise: a simple mechanism of conversion between binary and graded response in autoregulated genes. Phys Rev E.

[CR31] Pájaro M, Alonso AA, Vázquez C (2015). Shaping protein distributions in stochastic self-regulated gene expression networks. Phys Rev E.

[CR32] Pájaro M, Alonso AA, Carrillo JA, Vázquez C (2016). Stability of stochastic gene regulatory networks using entropy methods. IFAC-PapersOnLine.

[CR33] Pájaro M, Alonso AA, Otero-Muras I, Vázquez C (2017). Stochastic modeling and numerical simulation of gene regulatory networks with protein bursting. J Theor Biol.

[CR34] Pájaro M, Otero-Muras I, Vázquez C, Alonso AA (2018). SELANSI: a toolbox for simulation of stochastic gene regulatory networks. Bioinformatics.

[CR35] Paulsson J (2004). Summing up the noise in gene networks. Nature.

[CR36] Paulsson J (2005). Models of stochastic gene expression. Phys Life Rev.

[CR37] Perthame B (2007). Transport equations in biology. Frontiers in mathematics.

[CR38] Perthame B, Ryzhik L (2005). Exponential decay for the fragmentation or cell-division equation. J Differ Equ.

[CR39] Shahrezaei V, Swain PS (2008). Analytical distributions for stochastic gene expressions. Proc Natl Acad Sci USA.

[CR40] Sherman MS, Cohen BA (2014). A computational framework for analyzing stochasticity in gene expression. PLoS Comput Biol.

[CR41] Thomas P, Popovic N, Grima R (2014). Phenotypic switching in gene regulatory networks. Proc Natl Acad Sci USA.

[CR42] Van Kampen NG (2007). Stochastic processes in physics and chemistry.

[CR43] Wallace EWJ, Gillespie DT, Sanft KR, Petzold LR (2012). Linear noise approximation is valid over limited times for any chemical system that is sufficiently large. IET Syst Biol.

